# Sex hormones differently regulate lipid metabolism genes in primary human hepatocytes

**DOI:** 10.1186/s12902-024-01663-9

**Published:** 2024-08-01

**Authors:** Lena Seidemann, Clara Paula Lippold, Carolin Marie Rohm, Julian Connor Eckel, Gerda Schicht, Madlen Matz-Soja, Thomas Berg, Daniel Seehofer, Georg Damm

**Affiliations:** 1 Department of Hepatobiliary Surgery and Visceral Transplantation, Clinic for Visceral, Transplant, Thoracic and Vascular Surgery, Leipzig University Medical Center, 04103 Leipzig, Germany; 2 Saxonian Incubator for Clinical Translation (SIKT), Leipzig University, 04103 Leipzig, Germany; 3 Division of Hepatology, Department of Medicine II, Leipzig University Medical Center, 04103 Leipzig, Germany

**Keywords:** Primary human hepatocytes, Sex differences, Hepatic steatosis, MASLD, Sex hormones, 17β-estradiol, Testosterone, Progesterone

## Abstract

**Background:**

Prevalence of metabolic dysfunction-associated steatotic liver disease (MASLD) is higher in men than in women. Hormonal and genetic causes may account for the sex differences in MASLD. Current human in vitro liver models do not sufficiently take the influence of biological sex and sex hormones into consideration.

**Methods:**

Primary human hepatocytes (PHHs) were isolated from liver specimen of female and male donors and cultured with sex hormones (17β-estradiol, testosterone and progesterone) for up to 72 h. mRNA expression levels of 8 hepatic lipid metabolism genes were analyzed by RT-qPCR. Sex hormones and their metabolites were determined in cell culture supernatants by LC-MS analyses.

**Results:**

A sex-specific expression was observed for *LDLR* (low density lipoprotein receptor) with higher mRNA levels in male than female PHHs. All three sex hormones were metabolized by PHHs and the effects of hormones on gene expression levels varied depending on hepatocyte sex. Only in female PHHs, 17β-estradiol treatment affected expression levels of *PPARA* (peroxisome proliferator-activated receptor alpha), *LIPC* (hepatic lipase) and *APOL2* (apolipoprotein L2). Further changes in mRNA levels of female PHHs were observed for *ABCA1* (ATP-binding cassette, sub-family A, member 1) after testosterone and for *ABCA1*, *APOA5* (apolipoprotein A-V) and *PPARA* after progesterone treatment. Only the male PHHs showed changing mRNA levels for *LDLR* after 17β-estradiol and for *APOA5* after testosterone treatment.

**Conclusions:**

Male and female PHHs showed differences in their expression levels of hepatic lipid metabolism genes and their responsiveness towards sex hormones. Thus, cellular sex should be considered, especially when investigating the pathophysiological mechanisms of MASLD.

**Supplementary Information:**

The online version contains supplementary material available at 10.1186/s12902-024-01663-9.

## Background

Metabolic dysfunction-associated steatotic liver disease (MASLD) or non-alcoholic fatty liver disease (NAFLD) as it was named before [1], has become the most common cause of chronic liver disease in many parts of the world [2]. Its incidence and prevalence are higher in men than in women. Therefore, it has been coined a sexually dimorphic disease [3].

Hormonal and genetic causes may account for the sex differences in MASLD. Clinical observations suggest a strong influence of sex hormones (as mediators of the biological sex) on the development and progress of hepatic steatosis. Since MASLD incidence is higher in postmenopausal than in premenopausal women, but again lower in postmenopausal women on hormone replacement therapy, estrogen is deemed a protective factor in MASLD development [4, 5]. Likewise in men, deficiency of estrogen actions leads to hepatic steatosis, as can be observed in the rare cases of inactivating mutations of the aromatase or of estrogen receptor alpha [6]. Androgen levels, however, seem to have opposite effects on steatosis development in both sexes. While fatty liver is induced by high androgen levels in women (as e.g. in women with polycystic ovary syndrome), it develops in men under androgen deprivation (as therapeutically given for prostate cancer) [7, 8]. The role of progesterone in MASLD development is unclear, but seems to be rather unfavorable. Increased progesterone levels are associated with insulin resistance and hepatic lobular inflammation [3, 9].

As main site for steroid metabolism, the liver regulates sex hormone activity and clearance [6]. In hepatic phase I metabolism, sex hormones are hydroxylated, reduced or oxidized to be then conjugated (mainly by glucuronidation or sulfation) in phase II metabolism [10]. Thus, the lipophilic steroids are converted into more hydrophilic metabolites to enable urinary and biliary excretion [6, 10]. But interactions between sex hormones and the liver are not monodirectional. Apart from acting as a metabolic regulator, the liver is also a target organ of sex steroid signaling. Sex steroids regulate gene transcription by either directly acting as transcription factors and binding to hormone response elements on target genes (genomic mechanism) or by activating other transcription factors via downstream signaling pathways (non-genomic mechanisms) [11]. Transcriptional profiling of rodent liver tissues has established a genetic sexual dimorphism of the liver, indicating that certain genes show a higher expression level in males than females or vice versa [12, 13]. Receptors for all three sex hormones are expressed in male and female livers [9], and sex hormone receptor signaling is presumed to be a driver of the sexually dimorphic hepatic gene expression [14]. Among the > 70% of the sex-specifically expressed hepatic genes, a large amount is associated with lipid metabolism [15]. Few studies have targeted sex-specific differences in gene expression in the human liver. But those who have done so, have also found a pronounced sexual dimorphism in genes involved in lipid metabolism [16, 17].

So far, evidence on the role of sex and sex hormones in MASLD development is largely observational or based on preclinical studies mainly done in rodents. There is a lack of human studies addressing this issue [6] and the implementation of in vitro models was suggested to tackle this complex task [11]. As cell lines have only one sex, their use in sex-specific investigations is limited. Primary human hepatocytes (PHHs) are considered the gold standard for in vitro models of hepatic metabolism [18]. This study was conceptualized to examine the influence of sex and sex hormones on the expression of hepatic genes involved in lipid metabolism in PHHs. The target genes were chosen from two studies reporting on sex-dimorphic gene expression in human liver tissues and are thus assumed to show a sex-biased expression [16, 17]. PHHs were isolated from liver tissues after hepatic resection from donors without or at maximum mild steatosis. Gene expression profiles were sex-specifically analyzed directly after cell isolation and after cell culture with addition of either 17β-estradiol, progesterone or testosterone. Furthermore, liquid chromatography–mass spectrometry (LC-MS) of cell culture supernatants was performed to evaluate the sex hormone metabolism during cell culture. Thus, our investigations give further insight into the molecular background of hepatic sexual dimorphism and the suitability of PHHs for research in this regard.

## Methods

### Cell isolation

Liver tissues for PHH isolation were obtained from patients undergoing hepatic resections at Leipzig University Hospital. The study was conducted in accordance with the principles of the Declaration of Helsinki and approved by the Ethics Committee of the Medical Faculty of Leipzig University (registration number 322/17-ek, date 2020/06/10, ratified on 2021/11/30 and registration number 425/21-ek, date 2021/11/30). All patients gave their informed consent. Detailed donor data are reported in Table 1. PHHs were isolated from macroscopically tumor-free tissue samples by a two-step EGTA/collagenase perfusion technique as described previously [19]. After isolation, the cell suspension was washed with phosphate buffered saline (PBS; Gibco, Paisley, UK). Viable cells were counted with the trypan blue exclusion technique using a Neubauer counting chamber. 5 × 10^6^ cells were centrifuged for 5 min at 4 °C and 51 x *g*. The cell pellet was lysed by adding 1 ml RNA-Solv reagent (VWR International GmbH, Darmstadt, Germany), the suspension was transferred to a sterile RNAse-free tube and stored at -80 °C for later RT-qPCR analyses. All other cells were resuspended in PHH culture medium (William’s Medium E with GlutaMAX™ (WME; Gibco), supplemented with 10% fetal bovine serum (FBS) superior (Sigma-Aldrich, St. Louis, MO, USA), 15 mM HEPES, 1% nonessential amino acids (MEM NEAA 100x), 1 mM sodium pyruvate, 100 U/100 µM penicillin/streptomycin (all provided by Gibco), 40 U/ml insulin (Eli Lilly and Company, Indianapolis, USA and 1 µg/ml dexamethasone (JENAPHARM, Jena, Germany)) and used for cell culture.

**Table 1:** Donor data

Donor	Sex	Age	BMI [kg/m^2^]	Steatosis^1^	Diagnosis
FD1	Female	24	26	None	Echinococcosis
FD2	Female	45	19	None	FNH
FD3	Female	45	28	5%	Hemangioma
FD4	Female	46	23	None	Sarcoma metastasis
FD5	Female	60	22	None	CRLM
FD6	Female	72	20	5%	iCCA
FD7	Female	65	22	None	Sarcoma metastasis
MD1	Male	38	28	< 1%	NET metastasis
MD2	Male	39	20	5%	Echinococcosis
MD3	Male	45	21	None	CRLM
MD4	Male	47	31	None	Liver abscess
MD5	Male	57	23	5%	CRLM
MD6	Male	65	25	1%	CRLM
MD7	Male	66	25	10%	CRLM

^1^As reported in the postoperative pathohistological examination

*Abbreviations* BMI, body mass index; FNH, focal nodular hyperplasia; CRLM, colorectal liver metastasis; iCCA, intrahepatic cholangiocellular carcinoma; NET, neuroendocrine tumor

### Cell treatment

**Fig. 1:**
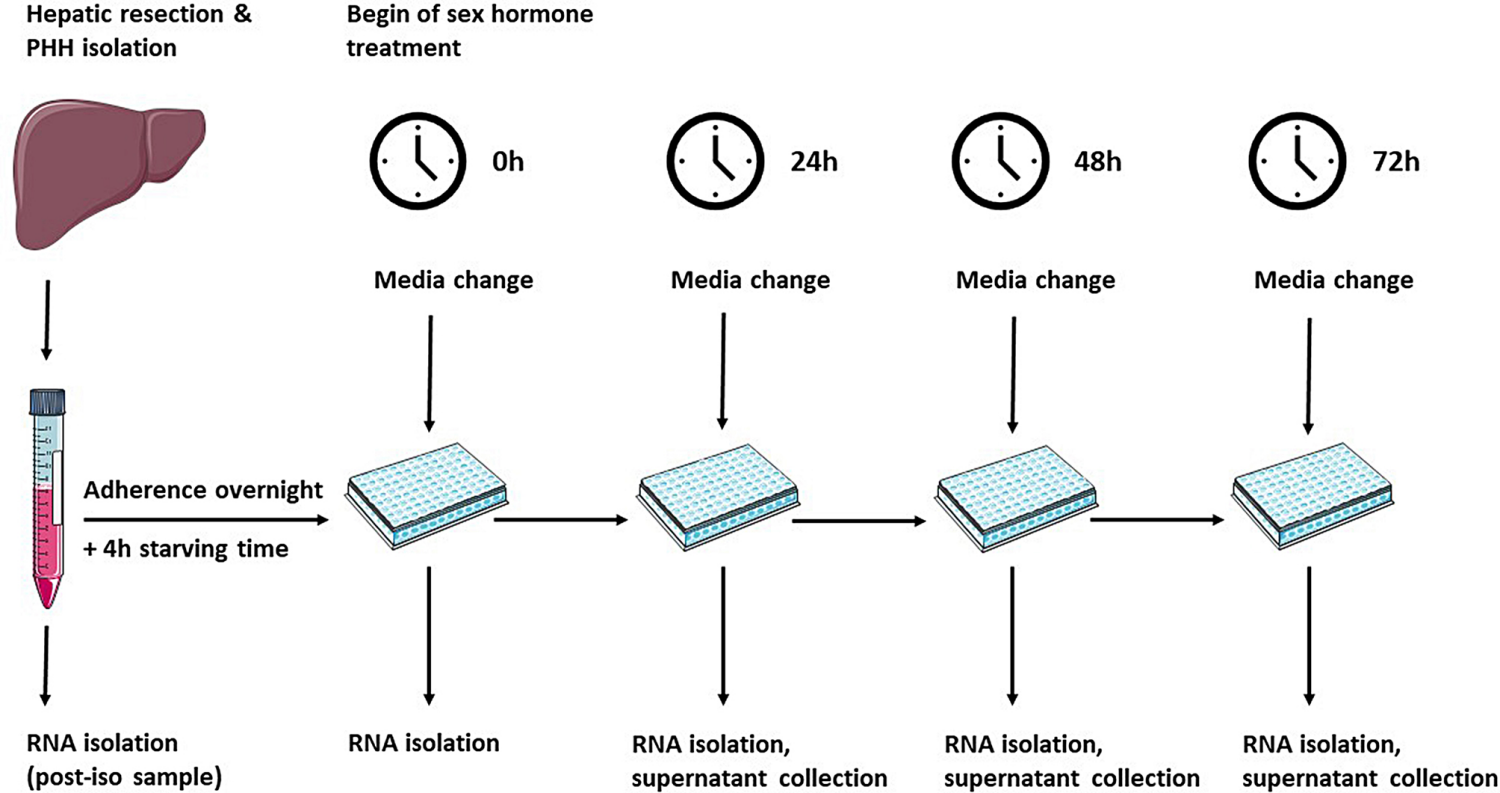
Schematic diagram of the experimental design for RNA analyses

Adherent cell cultures were used for RNA analyses. Cell culture dishes were coated with extracellular matrices based on collagen type I supplemented with 5% phenol-red free Matrigel (Corning Inc., Corning, NY, USA) as was described before [20]. In each of the wells of a 24 well plate, 380,000 PHHs homogenized in PHH culture medium were seeded. Four hours later, cells were washed with PBS to discard any debris and non-attached cells, and replenished with fresh PHH culture medium. After an overnight adherence phase at 37 °C with 5% CO_2_, cells were washed twice with PBS and the medium was changed to PHH starving medium (PHH culture medium on the basis of phenol-red free WME and without FBS, dexamethasone and insulin) in order to reduce effects from (growth) hormones. Following a 4 h “starving time”, the sex hormone treatment started. A schematic diagram of the experimental design is depicted in Fig. 1. PHHs were washed twice with PBS and the medium was changed to either PHH starving medium (control) or PHH starving medium supplemented with 10 nM 17β-estradiol, 70 nM progesterone, or 40 nM testosterone. Hormone concentrations were selected to correspond to the upper range of physiological human serum concentrations [21–23]. Sex hormones were obtained from Sigma-Aldrich (St. Louis, MO, USA; product no. E2758, P8783 and T1500) and dissolved in dimethyl sulfoxide (DMSO; Carl Roth, Karlsruhe, Germany), followed by dissolution in PHH starving medium to the desired concentration, resulting in a final DMSO concentration of 0.05%. Every 24 h, media including hormone or vehicle were changed and supernatants were collected and stored at -80 °C for later use in the LC-MS analyses. Beginning at 0 h and then every 24 h, PHHs were harvested for later RT-qPCR analyses after supernatant collection: cells were removed and lysed with a cell scraper after adding 200 µl RNA-Solv reagent to each well. The suspension was collected in sterile RNAse-free tubes and stored at -80 °C.

To determine cell activity throughout the course of cell culture, XTT assays (Cell Proliferation Kit II (XTT; Hoffmann-La Roche AG, Basel, Switzerland) were performed every 24 h on cells from 7 donors as described before [20].

For further investigations of hormone metabolism, suspension cell cultures from cryopreserved PHHs were used. Cryopreserved PHHs were obtained from our own biobank. PHH isolation was performed as prescribed above. Cells of three female (FD8-FD10) and three male donors (MD8-MD10) were thawed, PHH culture medium was added and cells were centrifuged for 5 min, 51 x g at room temperature and resuspended in PHH culture medium (detailed donor data are listed in Additional file 1: Table S1). Viable cells were counted with the trypan blue exclusion technique using a Neubauer counting chamber. Mean cell viability was 50% for female and 55% for male donors. Then, 2 × 10^6^ viable cells of each PHH donor were pooled for a female and a male specific batch. 0.5 × 10^6^ viable cells of each cell batch were treated with 50 µM 17β-estradiol, progesterone and testosterone, respectively. Untreated cells were used as controls. Cells were cultivated on a rotary incubator for 5 h at 37 °C and 5% CO_2_. Every hour, cells were removed from the incubator and briefly aerated under the sterile bench to allow gas exchange. The incubation was stopped by freezing at -80 °C, thawing and ultrasonic treatment for 5 min.

### RNA isolation, reverse transcription and qPCR analyses

The cell suspensions lysed in RNA-Solv reagent were thawed and subjected to the single-step method of RNA isolation as described by Chomczynski und Sacchi [24]. The resulting RNA pellet was resuspended in RNAse-free water (Qiagen, Venlo, Netherlands). RNA purity and integrity were determined with a NanoDrop 2000 (Thermo Fisher Scientific, Waltham, MA, USA). Reverse transcription was performed with the QuantiNova or QuantiTect Reverse Transcription Kits provided by Qiagen, following the manufacturer’s instructions. Specific primers for mRNA quantification were either purchased from Qiagen or gene-specific intron-spanning primers were designed using Primer3 software. Primer specifications are listed in Additional file 1: Table S2. Primer efficiencies were calculated from slopes of cDNA serial dilution Cq values. Primer specificity was confirmed by melt curve analyses and gel electrophoresis. qPCR analyses were performed in duplicate with 20 ng cDNA per reaction using the QuantiNova^®^ SYBR^®^ Green PCR Kit (Qiagen) with the 7500 Real-Time PCR System and the software v2.0.6. (Applied Biosystems, Foster City, CA, USA). The applied cycling profile was: 5 min initial heat activation at 95 °C and 10 s denaturation at 95 °C, followed by 30 s combined annealing/extension at 60–62 °C for 40 cycles. *RPL13A*, *EEF2* and *RPS18* served as reference genes. Relative gene expression was calculated according to the method described by Taylor et al. [25].

### Sample preparation for sex hormone metabolite analyses

The frozen cell culture supernatants from adherent cell cultures were thawed and centrifuged at 4500 rpm at 4 °C (Centrifuge 5424R, Eppendorf, Germany), 250 µl zinc sulfate solution 0,1 M (Sigma Aldrich, St. Louis, MO, USA) was added and vortexed for 20 s. Methanol (LC-MS grade; Carl Roth, Kalsruhe, Germany) was pre-cooled on ice and added into the supernatants which were then gently shaken on an incubator (Thermo compact 5350, Eppendorf, Hamburg, Germany) for 1 min to precipitate proteins. The samples were centrifuged at 8000 x g for 5 min, the supernatants were collected and the samples were spiked in a concentration of 50 nmol/l with their respective isotope internal standard. Here, progesterone-d9, testosterone-d3 and β-estradiol-d5 were used (provided in a concentration of 100 µg/ml acetonitrile by Merck, Darmstadt, Germany). Solid phase extraction columns (Chromabond, 730934) for steroid hormone extraction were placed on a vacuum manifold (both Macherey Nagel, Dueren, Germany) and conditioned in three separate steps with 1 ml acetonitrile, 1 ml methanol and 500 µl water with a vacuum of 0.9 bar (Vacuum pump, 22AN18, KNF Neuberger GmbH, Freiburg-Munzingen, Germany). The columns were loaded with 250 µl water and 1 ml sample and were eluted until dry. Then, the columns were washed with 500 µl water and in a second washing step with 500 µl water: methanol 7:3 v: v. The steroids were eluted by adding three times 500 µl and one time 250 µl acetonitrile.

Samples from the suspension cell cultures were prepared as follows: An aliquot of each sample was used for enzymatic cleavage of phase II metabolites. The samples were centrifuged at 1000 x g for 2 min. 0.15 M acetate buffer (acetic acid 9 g/l, 14,72 g/l potassium acetate, both provided by Carl Roth, Karlsruhe, Germany) pH 5 and 0.5 U/ml sulfatase from *helix pomatia* (Sigma-Aldrich, St. Louis, Missouri, United States) were added to the supernatants and incubated for 3 h at 37 °C in a thermo shaker. For metabolite extraction, all samples were extracted with 3 × 500 µl ethyl acetate (Carl Roth, Karlsruhe, Germany). Between each extraction step, samples were centrifuged at 10000 x g for 2 min. The collected organic phase was dried in a vacuum centrifuge at 45 °C for 2 h with 0.5 mTorr (Speedvac SPD 1030, Savant, Thermo Fisher Scientific, Waltham, Massachusetts, United States).

Thus prepared samples from supernatants or suspension cultures were dried in the vacuum centrifuge for 2 h at 34 °C and 5,1 mTorr. Dried samples were reconstituted in 50 µl eluent (50:50 water: acetonitrile) by 20 s vortexing. Then, each sample was transferred to glass vials with inserts.

### Liquid chromatography-mass spectrometry analyses

For analysis of the supernatants and suspension culture samples, liquid chromatography coupled mass spectrometry was used. The ultra performance liquid chromatography (UPLC) system for the analysis was an Aquity UPLC H class equipped with a binary pump, vacuum solvent degasser, column oven and autosampler. The mass spectrometry system applied was a Xevo XS qTof with an electrospray ion source (ESI, mass accuracy +/- 0.001) controlled by massLynx 4.1 software (all from Waters, Milford, MA, USA). The chromatographic separation was performed on a C18 Acquitiy UPLC column (1.7 μm 2,1 × 100 mm) equipped with an in-line filter and column temperature was set to 40 °C. The flow rate was set to 300 µl/min. The mobile phase consisted of A: water with 0.05 mM formic acid and 0.05 mM NH_4_Cl and B: pure acetonitrile. A gradient program was used (Additional file 1: Table S3). Sample injection volume was 10 µl. The ESI source was run on both positive and negative modes. Spectrometry parameters are listed in Additional file 1: Table S4.

### Data analysis and statistics

Statistical analyses of the qPCR experiments were performed with the software GraphPad Prism 8.0.2. Normality testing was performed with the Shapiro-Wilk test to decide for consecutive parametric or non-parametric testing. Differences between independent groups were analyzed with unpaired t test (with Welch’s correction, if variances were significantly different) or Mann-Whitney test as appropriate. Differences between paired samples were analyzed with paired t test or Wilcoxon test as appropriate. Mass spectrometry data were analyzed with massLynx 4.1 software (Waters, Milford, MA, USA). In brief, mass-specific traces were extracted from chromatograms. Corresponding peaks were integrated to obtain areas under the curve (AUC) values.

## Results

### Sex-specific gene expression after isolation and during cell culture without sex hormone treatment

**Fig. 2:**
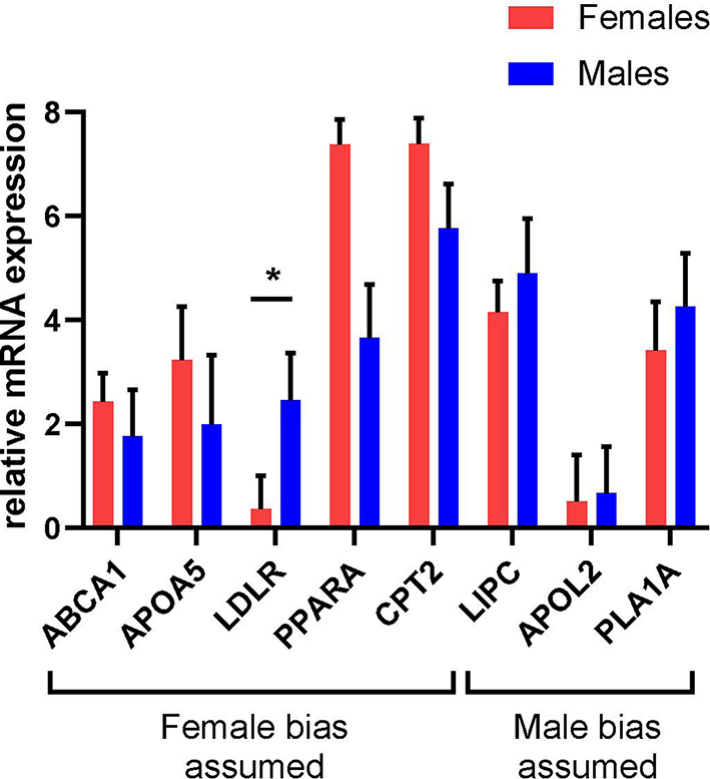
Sex-specific mRNA expression levels of primary human hepatocytes (PHHs) immediately after isolation from liver tissues of female and male donors. mRNA expression levels of ATP-binding cassette, sub-family A, member 1 (*ABCA1*), apolipoprotein A-V (*APOA5*), low density lipoprotein receptor (*LDLR*), peroxisome proliferator-activated receptor alpha (*PPARA*), carnitine palmitoyltransferase 2 (*CPT2*), hepatic lipase (*LIPC*), apolipoprotein L2 (*APOL2*) and phospholipase A1 member A (*PLA1A*) were analyzed by RT-qPCR. The assumed sex bias of the analyzed genes according to literature references [16, 17] is indicated below. Data are shown as geometric mean fold change (FC) + SEM, *n* = 7 per sex, *p* < 0.05 (*). For a visualization of individual FC values per donor see additional file 1: Fig. S2

**Fig. 3:**
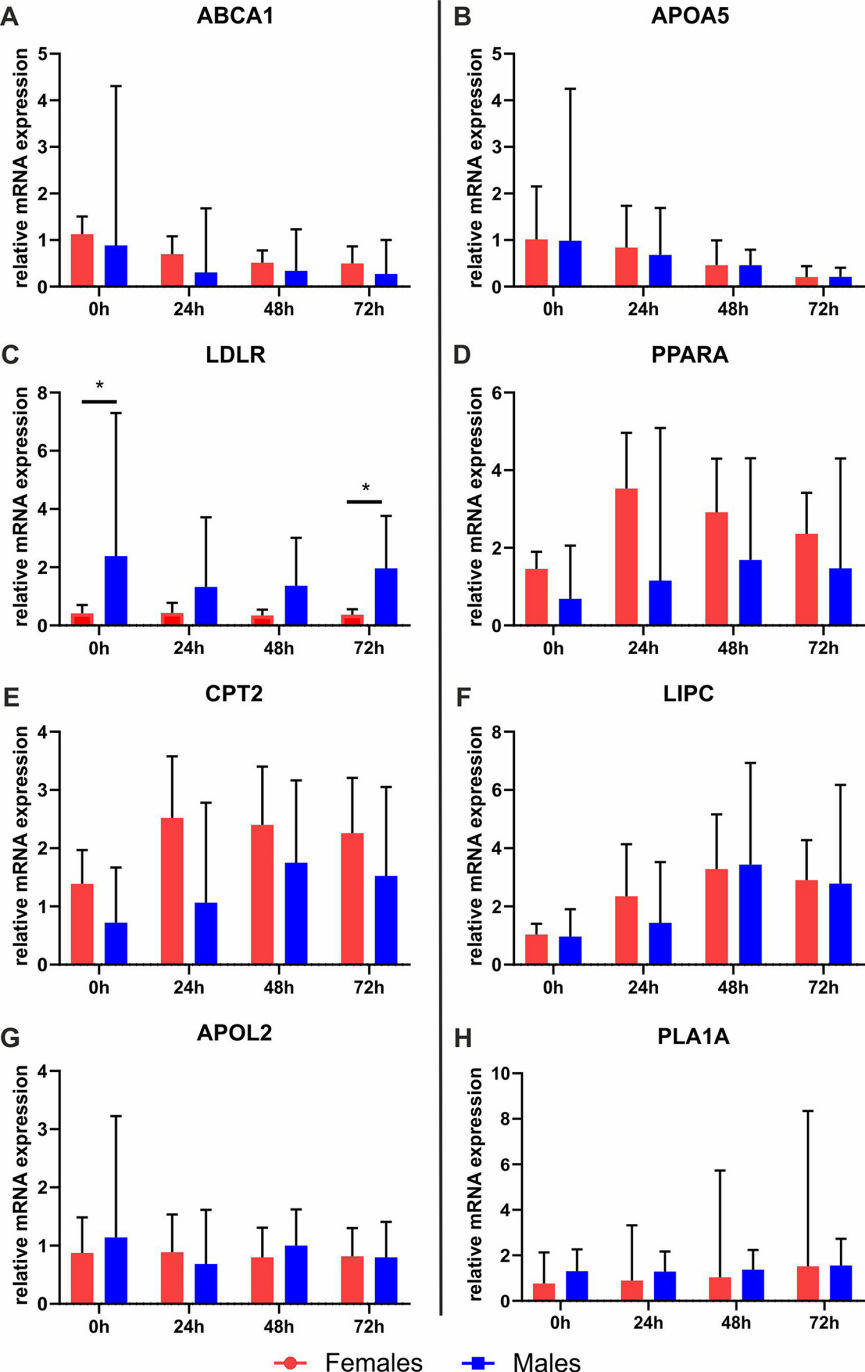
Sex-specific gene expression of primary human hepatocytes (PHHs) during cell culture without sex hormones. PHHs were isolated from liver tissues of female and male donors, cultured with PHH starving medium for up to 72 h and mRNA expression levels of **A** ATP-binding cassette, sub-family A, member 1 (*ABCA1*), **B** apolipoprotein A-V (*APOA5*), **C** low density lipoprotein receptor (*LDLR*), **D** peroxisome proliferator-activated receptor alpha (*PPARA*), **E** carnitine palmitoyltransferase 2 (*CPT2*), **F** hepatic lipase (*LIPC*), **G** apolipoprotein L2 (*APOL2*) and **H** phospholipase A1 member A (*PLA1A*) were analyzed by RT-qPCR. Data are shown as the geometric mean fold change + SEM, *n* = 7 per sex, *p* < 0.05 (*)

The mRNA expression levels of 8 genes, for which a sex-biased expression in liver tissues was reported [16, 17], were analyzed by RT-qPCR directly after isolation and during adherent cell culture in PHHs of female or male origin. In the literature, it was assumed that five of the analyzed genes show higher expression in female PHHs: ATP-binding cassette, sub-family A, member 1 (*ABCA1*), apolipoprotein A-V (*APOA5*), low density lipoprotein receptor (*LDLR*), peroxisome proliferator-activated receptor alpha (*PPARA*) and carnitine palmitoyltransferase 2 (*CPT2*). For *PPARA* and *CPT2*, mRNA expression values were higher by trend in female PHHs directly after isolation as well as during cell culture for up to 72 h (Fig. 2; additional file 1: Fig. S2, Table S5). A significantly different expression between PHHs of different sex was only observed for *LDLR*, which showed a male bias in contrast to the initial assumption. For the supposedly male biased genes hepatic lipase (*LIPC*), apolipoprotein L2 (*APOL2*) and phospholipase A1 member A (*PLA1A*), no clear sex difference could be observed. Overall, all analyzed genes showed stable expression levels over the course of 72 h under the applicated culture conditions (Fig. 3). Furthermore, stable cell viability over the course of 72 h with and without sex hormone treatment was confirmed by XTT assay (Additional file 1: Fig. S1).

### Sex hormones were metabolized in sex-specific PHH cultures

**Fig. 4:**
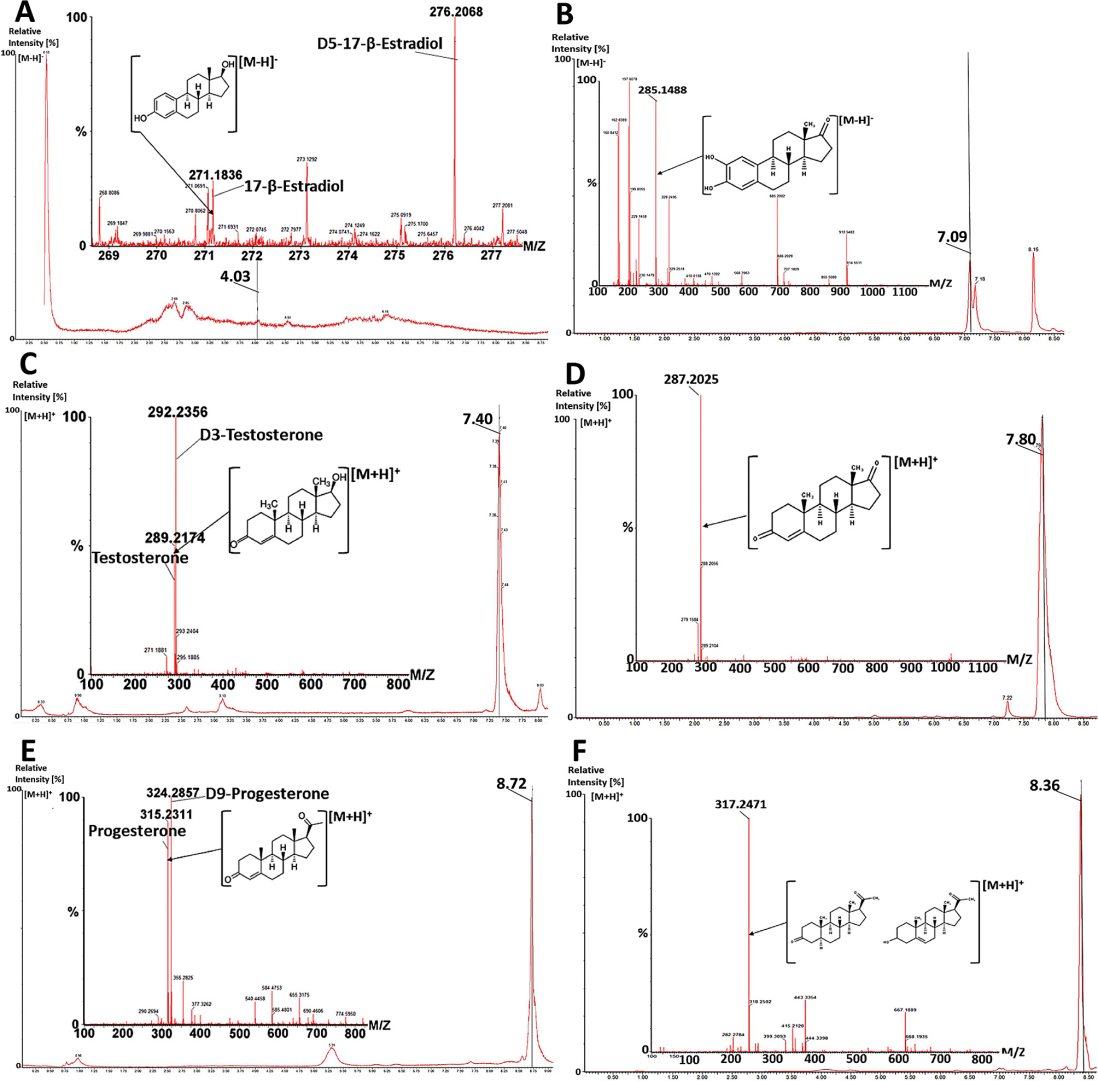
Chromatograms and mass spectra of **A** 17β-estradiol, **C** testosterone, **E** progesterone and their isotope controls with their main metabolites **B** 2-hydroxyestrone, **D** androstenedione and **F** dihydroxyprogesterone / pregnenolone in the respective medium before (**A**, **C**, **E**) and after (**B**, **D**, **F**) cell culture

The stability of sex hormones in adherent PHH cultures was investigated by LC-MS analysis. For that, the respective hormones were quantified before (Fig. 4) and after an incubation time of 24 h (Table 2). Hormones were identified after enzymatic cleavage of their phase II metabolites by their specific mass (Fig. 4). Analysis of the cell culture supernatants after 24 h revealed that applied hormone concentrations decreased by 96–100% in all donors (Table 2) suggesting the hormones were metabolized.

For reference, we generated hormone-specific metabolite patterns by treating PHHs in suspension cultures with the respective hormones. For receiving clear metabolite peaks we chose higher concentrations of the respective hormones and shorter incubation times in comparison to our adherent PHH cultures. Metabolites were identified by their accurate masses (Table 3).

After PHH incubation with 17β-estradiol, the parent ion (C18H24O2; [M-H]- = 271.1836) vanished completely. Since 17β-estradiol was poorly ionizable, the complete loss we observed, allows no conclusion of 17β-estradiol’s metabolism rate. A main metabolite (m/z = 285.1488) and a further metabolite (m/z = 287.1645) were detected in male and female culture samples. Two metabolites (m/z = 271.1713 and m/z = 301.1815) were detected only in female cultures. The retention times of all metabolites were later than that for 17β-estradiol (3.97) indicating metabolites with a lower polarity than 17β-estradiol No metabolite ions were detected in control samples (PHH cell culture medium with and without 17β-estradiol prior to cell culture and female/male cells without added hormones).

After PHH incubation with testosterone, the concentration of testosterone (parent ion; C19H28O2; MH + = 289.2209) was reduced by around 28 µM in female and around 20 µM in male PHH cultures indicating a higher metabolic activity in the female PHH pool. A main metabolite (m/z = 287.2059) and three minor metabolites (m/z = 303.1949; m/z = 305.2115, m/z = 291.2324) were formed in male and female cultures. The retention time of the main metabolite was 0.3 min later than that of testosterone (7.8 min vs. 7.5 min) and the metabolite m/z = 291.2324 was 0.65 min later than that of testosterone (8.15 min vs. 7.5 min) indicating metabolites with a lower polarity than testosterone. In contrast, the two metabolites m/z = 303.1949 and m/z = 305.2115 were detected at lower retention times than testosterone indicating metabolic transformations leading to more polar structures. No metabolite ions were detected in respective controls.

Progesterone showed a very low metabolism rate in suspension cultures of both sexes. After PHH incubation with progesterone, the concentration of progesterone (parent ion; C21H30O2; MH + = 315.2347) was reduced by around 8.5 µM in female, while in male PHH no reduction was noticeable. However, a main metabolite (m/z = 317.2471) and one further metabolite (m/z = 331.2263) were formed in male and female cultures. The retention time of the main metabolite was 0.43 min earlier than that of progesterone (8.38 min vs. 8.81 min) and the metabolite m/z = 331.2263 was 1.13 min earlier than that of progesterone (7.68 min vs. 8.81 min) indicating metabolites with a higher polarity than progesterone. Metabolite ions were not detected in the respective controls.

The high accuracy of the detected masses in combination with the shifts in retention times of detected peaks allowed the allocation to metabolites of the respective hormones known from the literature [26–30] (Table 3). Taken together, our metabolism experiment revealed that primary oxidation as well as hydroxylation reactions take place in cultured PHHs. The search for respective reference hormone metabolite masses in supernatants of adherent cultures neither revealed phase II metabolites of the respective hormones nor the phase I metabolites found in our suspension cultures or their respective phase II metabolites. However, treatment of adherent PHH cultures with 17β-estradiol, testosterone and progesterone for up to 72 h, led to a sex- and hormone-specific induction of sex hormone metabolizing enzymes *CYP3A5* (Cytochrome P450 3A5), *UGT2B15* (UDP-glucuronosyltransferase 2B15) and *SULT1A1* (sulfotransferase family 1 A member 1) (Additional file 1: Fig. S4-6).

**Table 2:** LC-MS analytics of sex hormones in adherent PHH cultures

Sample	AUC of E2	Metabolized E2 after 24 h [%]	AUC of T	Metabolized T after 24 h [%]	AUC of *P*	Metabolized *P* after 24 h [%]
NC	419	-	29,667	-	46,289	-
FD1	ND	100	6441	96.9	3071	99.6
FD2	ND	100	3994	98.3	1782	99.8
FD3	ND	100	2349	98.8	1223	99.8
FD4	ND	100	3327	98.5	895	99.9
FD5	ND	100	2762	98.4	1004	99.8
MD1	ND	100	5983	98.0	562	99.9
MD2	ND	100	2870	98.8	2101	99.7
MD3	ND	100	5216	98.0	787	99.9
MD4	ND	100	2272	98.8	730	99.9
MD5	ND	100	3413	98.3	868	99.9

Primary human hepatocytes (PHHs) were cultured with one of the respective sex hormones for 24 h. Cell culture media prior to cell culture were used as negative controls. Values represent integrated peak areas (area under the curve; AUC) for the parent of the respective hormone normalized to the AUC of its respective isotopic standard. The amount of hormone metabolized after 24 h is given as percentage of the respective hormone value in relation to its negative control value

*Abbreviations* E2, 17β-estradiol; T, testosterone; P, progesterone

**Table 3:** Overview of metabolite patterns in PHH suspension cultures

Treatment	Formular	Theoretical [m/z]	Measured [m/z]	Speculated metabolite	Retention time [min]	Female [µmol/l]	Male [µmol/l]
17β-estradiol	C18H24O2	271.1698 [M-H]^−^	271.1635	17β-estradiol	3.97	ND	ND
	C18H22O2	271.1698 [M + H]^+^	271.1713	Estrone	7.79	10.70	ND
	C19H24O3	301.1804 [M + H]^+^	301.1815	2-Methoxy-estrone	8.03	1.65	ND
	C18H24O3	287.1647 [M-H]^−^	287.1645	2-Hydroxy-estradiol	6.59	13.69	6.57
	C18H22O3	285.1491 [M-H]^−^	285.1488	2-Hydroxy-estrone	7.07	55.08	10.05
Testosterone	C19H28O2	289.2168 [M + H]^+^	289.2209	Testosterone	7.50	21.97	29.49
	C19H28O3	305.2117 [M + H]^+^	305.2115	Hydroxy-testosterone	5.37	0.71	0.59
	C19H26O3	303.1960 [M + H]^+^	303.1949	11-Keto-testosterone	5.72	0.98	0.50
	C19H30O2	291.2324 [M + H]^+^	291.2324	Dihydro-testosterone	8.15	0.16	0.047
	C19H26O2	287.2011 [M + H]^+^	287.2059	Androstene-dione	7.80	27.75	29.26
Progesterone	C21H30O2	315.2324 [M + H]^+^	315.2347	Progesterone	8.81	41.54	51.48
	C21H32O2	317.2481 [M + H]^+^	317.2471	Dihydro-progesterone/ Pregnenolone	8.38	4.06	4.38
	C21H30O3	331.2273 [M + H]^+^	331.2264	Hydroxy-progesterone	7.68	0.83	0.53

Primary human hepatocytes (PHHs) from female and male donors were cultured with 50 µM of one of the respective sex hormones for 5 h. Cell culture media were collected and analyzed by LC-MS after cleavage of phase II metabolites. Metabolite amounts are expressed semi-quantitatively in relation to the concentration of the initially applied respective parent hormone. For chromatograms and mass spectra see Fig. 4 and Additional file 1: Fig. S3

### The influence of sex steroids on lipid metabolism genes depends on the sex of PHH donors

**Fig. 5:**
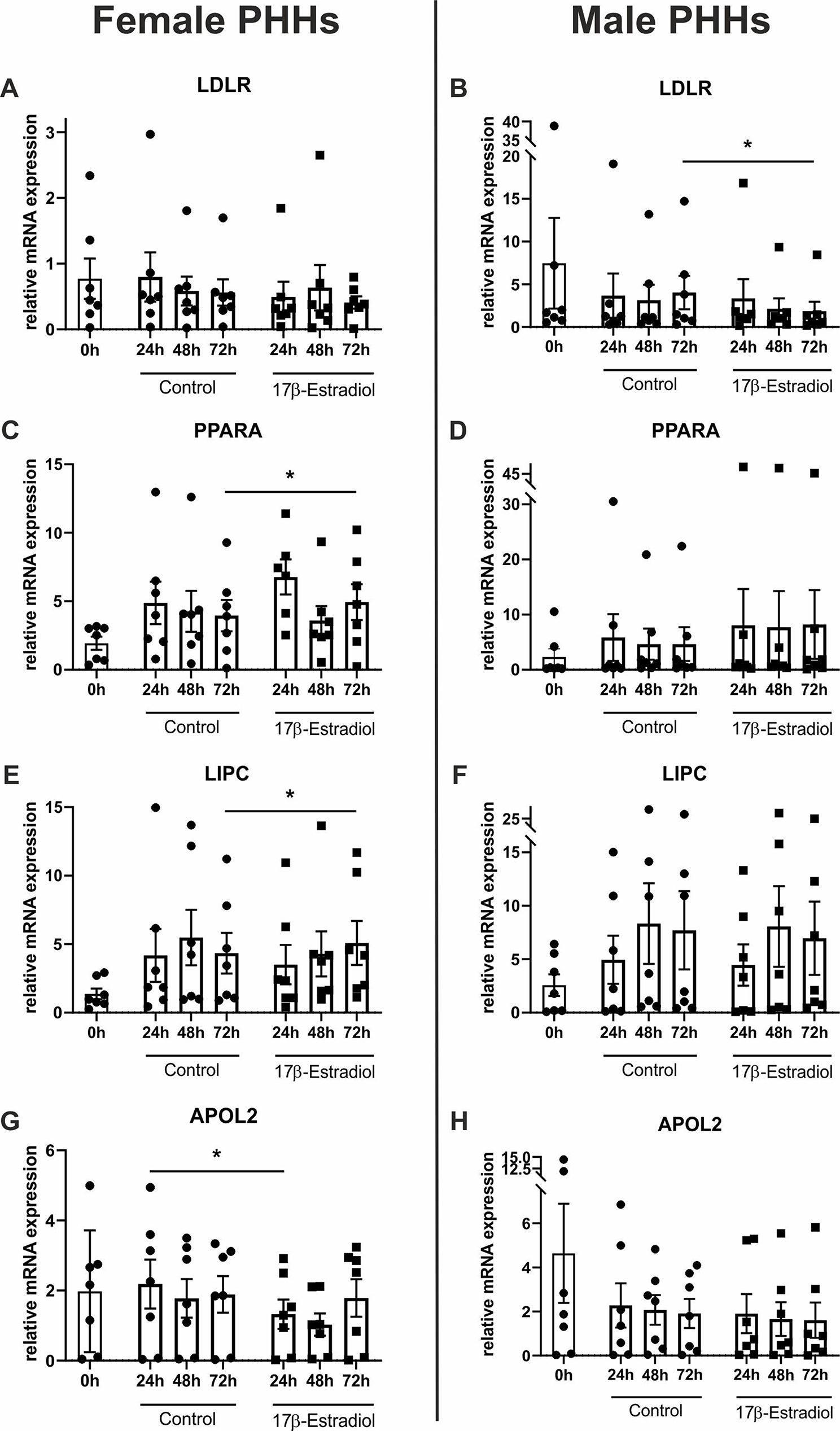
Influence of 17β-estradiol on mRNA expression levels of primary human hepatocytes (PHHs) of different sex. PHHs were isolated from liver tissues of female (**A**,** C**,** E**,** G**) and male (**B**,** D**,** F**,** H**) donors, cultured with PHH starving medium supplemented with 10 nM 17β-estradiol for up to 72 h and mRNA expression levels levels of **A**,** B** low density lipoprotein receptor (*LDLR*), **C**,** D** peroxisome proliferator-activated receptor alpha (*PPARA*), **C**,** D** hepatic lipase (*LIPC*) and **G**,** H** apolipoprotein L2 (*APOL2*) were analyzed sex-specifically by RT-qPCR. Individual fold change values are displayed as dots and cubes, bar graphs represent means ± SEM, *n* = 7 per sex, *p* < 0.05 (*)

**Fig. 6:**
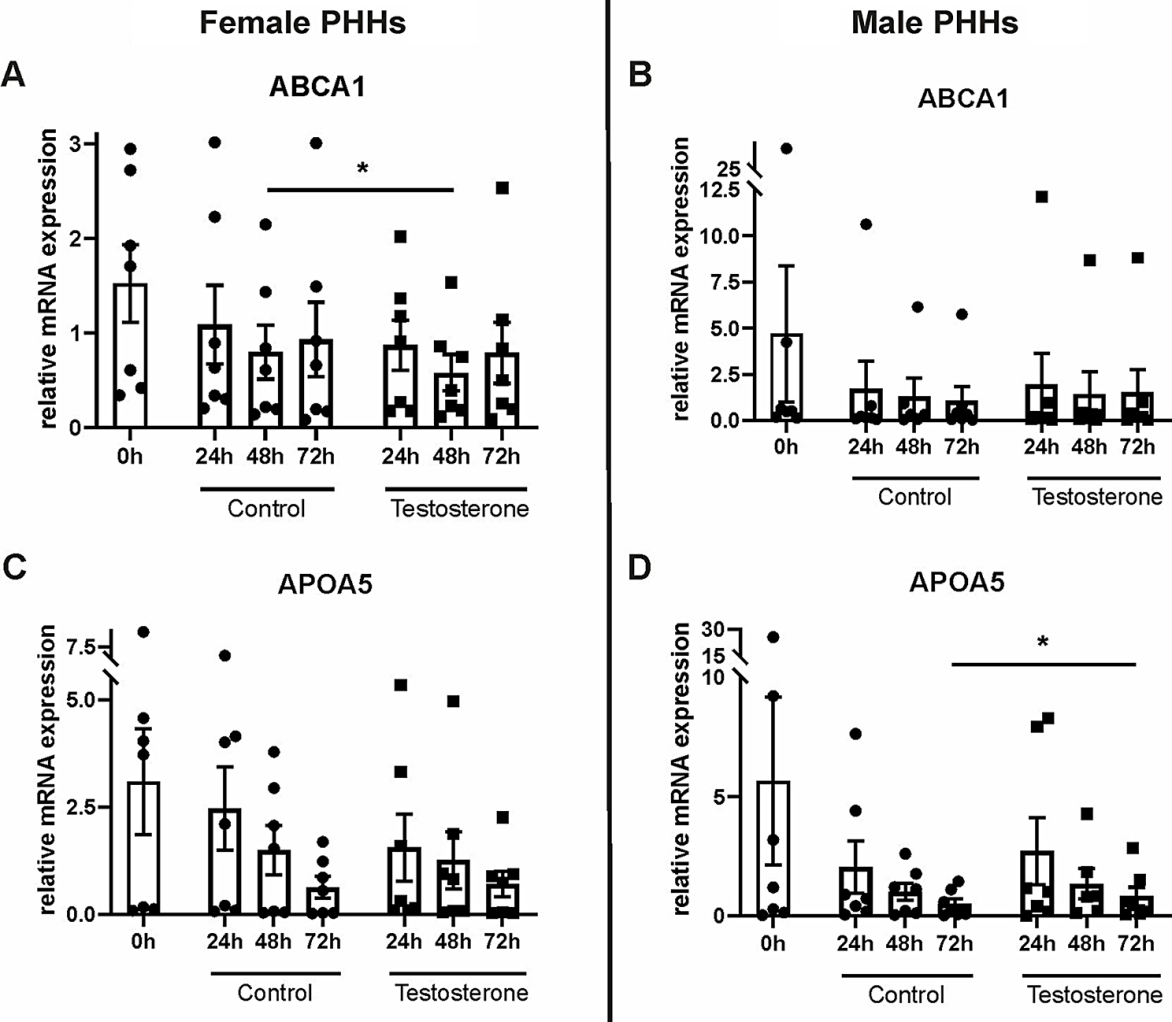
Influence of testosterone on mRNA expression levels of primary human hepatocytes (PHHs) of different sex. PHHs were isolated from liver tissues of female (**A**,** C**) and male (**B**,** D**) donors, cultured with PHH starving medium supplemented with 40 nM testosterone for up to 72 h and mRNA expression levels of **A**,** B** ATP-binding cassette, sub-family A, member 1 (*ABCA1*) and **C**,** D** apolipoprotein A-V (*APOA5*) were analyzed by RT-qPCR. Individual fold change values are displayed as dots and cubes, bar graphs display means ± SEM, *n* = 7 per sex, *p* < 0.05 (*)

**Fig. 7:**
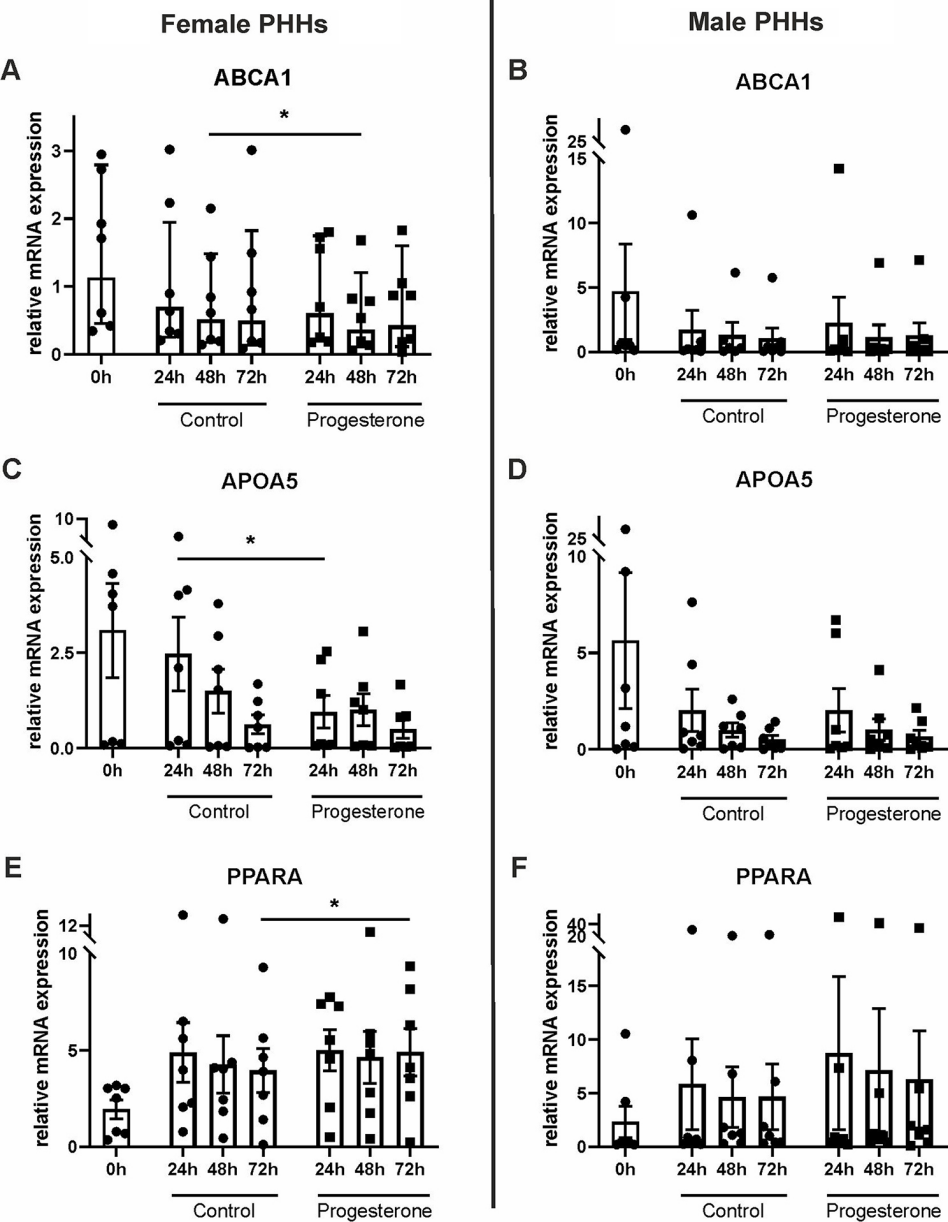
Influence of progesterone on mRNA expression levels of primary human hepatocytes (PHHs) of different sex. PHHs were isolated from liver tissues of female (**A**,** C**,** E**) and male (**B**,** D. F**) donors, cultured with PHH starving medium supplemented with 70 nM progesterone for up to 72 h and mRNA expression levels of **A**,** B** ATP-binding cassette, sub-family A, member 1 (*ABCA1*), **C**,** D** apolipoprotein A-V (*APOA5*) and **E**,** F** peroxisome proliferator-activated receptor alpha (*PPARA*) were analyzed by RT-qPCR. Individual fold change values are displayed as dots and cubes, bar graphs display means ± SEM, *n* = 7 per sex, *p* < 0.05 (*)

PHHs were cultured with physiological concentrations of the sex hormones 17β-estradiol, progesterone or testosterone for up to 72 h. The influence of these hormones on mRNA expression levels of supposedly sex-biased genes of the hepatic lipid metabolism was analyzed by RT-qPCR. Here, varying effects of the hormones on hepatocytes of different sex could be observed. 17β-estradiol treatment led to an increase of the mRNA levels of *PPARA* and *LIPC* only in PHHs of female origin (Fig. 5, Additional file 1: Table S6). Also, *APOL2* expression slightly decreased only in female PHHs at the beginning of the 17β-estradiol treatment. In male PHHs, only *LDLR* expression responded on 17β-estradiol treatment, which led to a significant decrease. Addition of testosterone to the cell culture medium increased the *APOA5* mRNA levels only in male and decreased the *ABCA1* mRNA levels only in female PHHs (Fig. 6, Additional file 1: Table S7). Analogously to the effect of 17β-estradiol, also progesterone increased the *PPARA* mRNA levels in PHHs from female donors. In contrast, *ABCA1* and *APOA5* mRNA levels were reduced, when female hepatocytes were treated with progesterone (Fig. 7, Additional file 1: Table S8). Concordant reductions in mRNA levels in PHHs from both sexes were observed after progesterone treatment for *APOL2*, *CPT2*, *LDLR* and *LIPC*. For *CPT2*, also testosterone significantly lowered the mRNA expression in hepatocytes from males and females (Additional file 1: Tables S7, S8)

## Discussion

Although it has been established that biological sex is an important variable in disease development and progression already on the cellular level, in vitro research that takes the sex of cells into account is very complex and still underrepresented [31]. Since MASLD is an increasing worldwide health threat and preclinical research covering the sexual dimorphism of the disease has largely been done in rodents, advancing the knowledge on the human level is especially desired. In this study, we took on the call for examining primary cells of different sexes and the influence of sex hormones [32] in the context of hepatic lipid metabolism.

First, we characterized the sex-dependent expression of lipid metabolism genes in PHHs. Basis for our analyses was a literature search on sex-biased gene expression in the human liver. We found two studies on this topic reporting degrees and directions of sex-biased genes involved in hepatic lipid metabolism that were suitable for our research purposes [16, 17]. Out of these we extracted eight genes with reported sex bias that are involved in hepatic lipid metabolism. Five genes with an assumed female bias (*ABCA1*, *APOA5*, *LDLR*, *PPARA*, *CPT2*) and three with an assumed male bias (*LIPC*, *APOL2*, *PLA1A*) were chosen for RT-qPCR analyses in PHHs from male and female donors. Compared to the microarray-based transcriptome data of Zhang et al. and García-Calzón et al. [16, 17] our targeted analyses of sex-specific mRNA expression levels immediately after hepatocyte isolation provided rather different results. For most of the genes with reported female bias, we could also see a trend in the same direction. This was especially the case for *PPARA*, a central transcription factor that regulates fatty acid beta oxidation. The relative *PPARA* mRNA expression was 7.37 in female hepatocytes, compared to 3.65 in male hepatocytes, although the difference was statistically not significant. A significant sex bias could however be observed for *LDLR* with a 6.8-times higher relative expression in male than female hepatocytes. This was in contrast to the higher *LDLR* expression in female liver tissues as reported by Zhang et al. [16], where however the male/female fold change was less pronounced with − 1.42. Unlike our analyses of hepatocytes only, the transcriptome study utilized liver biopsy specimens for mRNA analyses. Therefore, additionally to hepatocytes, non-parenchymal liver cells (NPCs) and blood cells (remaining in hepatic sinusoids) [33] have been included in the RNA isolation and subsequent analytical process. As for NPCs, especially hepatic stellate cells play an essential role in lipid metabolism [34]. Thus, analyses of whole tissue samples can result in a different sex bias on transcript level as compared to isolated hepatocytes. In this context, it is also important to state, that none of our hepatocyte donors was on lipid-lowering medication that could potentially induce *LDLR* mRNA expression like HMG-CoA (3-hydroxy-3 methylglutaryl coenzyme A) reductase inhibitors [35]. To the best of our knowledge, our data provide insights on hepatocyte-specific sex-biased mRNA expression in humans for the first time.

In the next step, we investigated the stability of mRNA expression of our sex-specific targets over a cultivation time of 72 h. We aimed to keep the bias introduced into our cell culture as low as possible. Therefore, serum and other hormone additives to the culture medium (e.g. phenol red, which is known to activate estrogen receptors) were excluded [31]. Under these conditions, our results show that all genes were stably expressed. A statistically significant sex difference could again only be observed for *LDLR*.

A downside of primary cell culture is the limited reproducibility due to inter-donor variability. Concerning our analyzed genes, we saw partly high variances at different time points. After the initial adherence phase, *ABCA1*, *APOA5*, *LDLR* and *APOL2* showed a high variance in male hepatocytes. Over the course of the culture, the individual expression levels equalize leading to smaller variations. It is known, that initially high read-out variations in PHHs can be explained by post-isolation stress. In contrast, larger variances at later time points during PHH culture can be explained by dedifferentiation [36]. The latter could explain the increasing variances observed for *LIPC* expression in both sexes and *PLA1A* expression in female hepatocytes. Taken together, our results suggest that effects of in vitro cultivation affect gene expression differently when comparing sexes. It is discussed controversially that missing systemic influences (e.g. hormones) during cell culture lead to dedifferentiation processes. The addition of hormones as an effective way of introducing a relevant sex specific element into cell models was suggested [31].

As was proposed by Mauvais-Jarvis and co-workers, we added sex hormones in physiological concentrations to the culture media [37]. Serum concentrations in humans range between 0.1 and 10 nM for 17β-estradiol, 5 and 70 nM for progesterone and 10 and 40 nM for testosterone [21, 22]. After preliminary experiments, we decided to apply the upper range of aforementioned concentrations. A four hours starving phase preceded the hormone treatment to eliminate remaining hormones from adherence phase media (e.g. in FBS). Our hormone analyses of the adherent PHH culture supernatants using LC-MS showed that > 95% of the added sex hormones were metabolized during 24 h cultivation time. The liver is known for its great capacity in xenobiotic metabolism, which also affects sex hormones. Our investigation of sex specific phase I sex steroid metabolism confirmed biotransformation of all three sex hormones. Metabolism of 17β-estradiol and testosterone was dominated by conversion to estrone or estrone metabolites and androstenedione, respectively. Both reactions lead to formation of less active steroids, which are catalyzed by 17ß-hydroxysteroid-dehydrogenase [38]. In addition, we detected hydroxylated metabolites of 17β-estradiol, estrone and testosterone. It is known that biotransformation of steroids by CYP450 isoenzymes leads to a multitude of different hydroxysteroids. While we detected hydroxy steroids of all three tested hormones, these played a minor part in testosterone and progesterone metabolite profiles. Estradiol is also metabolized via hydroxylation at the 2 or 4 position into their respective catechol estrogens [39] which are a substrate for subsequent methylation by catechol-O-methyl transferases. The fact that we detected methoxyestrone in our female samples indicates the formation of reactive catechol estrogens in female livers. Testosterone and progesterone were also transformed to their dihydro metabolites. Reduction of testosterone to dihydro testosterone and progesterone to dihydro progesterone are catalyzed by 5a-reductase [40]. This reaction was less pronounced in testosterone metabolism of our donors. In contrast, progesterone is prone to enzymatic reduction by reductases and hydroxysteroid dehydrogenases during hepatic metabolism [41]. While we observed dihydro progesterone as a main metabolite we were not able to detect the subsequently formed pregnanolone isomers. This may be due to the general low metabolic conversion rate of progesterone in our model system.

In our LC-MS analyses of the supernatants of the 24 h adherent PHH cultures, we were not able to detect the parent, our reference phase I metabolite patterns or their respective phase II metabolites. However, the induction of hormone metabolism enzymes *CYP3A5*, *UGT2B15* and *SULT1A1* on transcript level shows that the used hormone concentrations were sufficiently high to exert effects. All three enzymes are known to be central regulators of sex hormone deactivation and excretion [10]. The missing of metabolite traces suggests low metabolic conversion or formation of complexly modified metabolites. The latter could be due to the cultivation time of 24 h in combination with the low hormone concentrations, leading to repeated transformation processes.

**Fig. 8:**
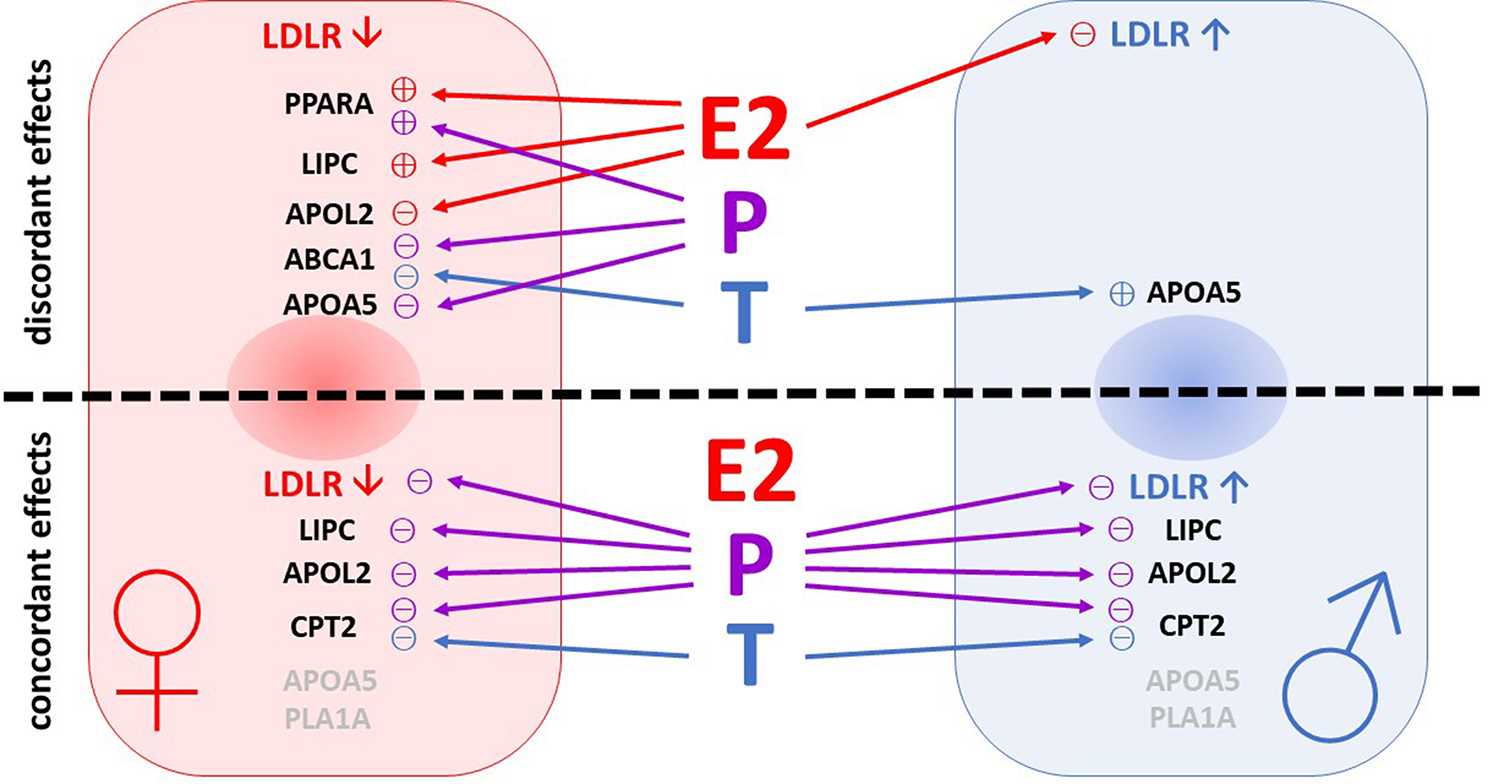
Overview of sex-related (indicated with arrow signs) and sex hormone-related (indicated with plus and minus signs) expression of lipid metabolism genes in primary human hepatocytes (PHHs) of female (left) and male (right) origin. Abbreviations: E2, 17β-estradiol; P, progesterone; T, testosterone; *LDLR*, low density lipoprotein receptor; *PPARA*, peroxisome proliferator-activated receptor alpha; *LIPC*, hepatic lipase; *APOL2*, apolipoprotein L2; *ABCA1*, ATP-binding cassette, sub-family A, member 1; *APOA5*, apolipoprotein A-V; *CPT2*, carnitine palmitoyltransferase 2; *PLA1A*, phospholipase A1 member A

After incubation with 17β-estradiol we observed significant effects on mRNA expression levels in 4, with progesterone in 7 and with testosterone in 3 of the 8 analyzed genes (for a comprehensive overview see Fig. 8). The high number of differentially expressed genes after progesterone treatment complies with its low metabolism rate seen in our LC-MS results. While most of the transcriptional effects mediated by progesterone were concordant in PHHs of both sexes, 17β-estradiol and testosterone effects differed more strongly between sexes. In this regard, the most striking effects were seen for *LDLR* and *PPARA*. The *LDLR* gene, which had a male biased expression in our results, showed reduced mRNA levels in male PHHs after cultivation with 17β-estradiol. Cultivation with the other female sex steroid progesterone reduced *LDLR* mRNA levels in PHHs of both sexes (see Additional file 1: Table S8). These results were surprising since a positive link between LDLR and female sex / estrogen is widely assumed. LDLR, the hepatic cell surface receptor for low density lipoprotein (LDL) is the major site for LDL cholesterol (LDLC) plasma clearance. Premenopausal women show lower LDLC plasma levels of in comparison to age-matched men or postmenopausal women. This has been attributed to an increased LDLR binding activity and protein expression in response to estrogen [42]. However, estrogen mediates its positive effects on LDLR by post-transcriptional regulation [42, 43]. Although also *LDLR* transcription has been shown to be estrogen-responsive, it seems that the application of supraphysiological hormone concentrations is necessary to exert this effect. In their study on the human hepatoma cell line Huh7, Starr et al. observed a transcriptional upregulation of *LDLR* only at the highest dose tested (10 µM), which was by the factor 1000 higher than the physiological concentration we applied (10 nM) [42]. On protein level, Starr and colleagues observed an upregulation already after treatment with 3 µM and the level of the protein expression observed after 10 µM 17β-estradiol treatment considerably exceeded that of the transcriptional regulation. Another study performed on human HepG2 hepatoma cells did also not observe transcriptional activation of *LDLR* after treatment with 10 and 100 nM 17β-estradiol [43]. The absence of a transcriptional activation of *LDLR* in our results may be due to the use of physiological hormone concentrations. Furthermore, androgens have been shown to oppose estrogen’s positive effects on *LDLR* transcription in HepG2 cells [44]. The negative effect of 17β-estradiol and progesterone on *LDLR* mRNA expression in male hepatocytes suggests that these react more sensitive when the hormone treatment is in contrast to the sex-specific environment. Also, for *PPARA* a positive association to female sex and gonadal hormones is described. In post-menopausal women and ovariectomized rodents, *PPARA* and its downstream targets of the fatty acid beta oxidation pathway are downregulated [45]. In the animal model the effects of ovariectomy could be prevented by estrogen replacement [46]. In line with these findings, we observed an upregulation of *PPARA* expression in female hepatocytes treated with 17β-estradiol and additionally with progesterone after 72 h. The female control hepatocytes displayed a slight decrease of *PPARA* expression over the cultivation time, although this was not statistically significant. Nonetheless, the effects of the female sex hormones point to a positive influence of the same sex cell culture environment. Taken together, our results affirm that donor sex matters when applying primary cells in in vitro models. Hormone treatment leads to rather a fine-tuning than to drastic changes in metabolic transcriptional regulation. Besides donor sex, other individual factors account for variations in basal gene expression levels as well as for levels of hormone responsiveness.

Utilizing primary cells, we have to accept interindividual variations leading to larger standard deviations in our experimental results as e.g. the use of cell lines would yield. Yet, PHHs reflect the physiological situation better than cell lines could do. Especially when the study of sex differences is the research objective, cell lines are no proper surrogate. Therefore, this study pursued the target to evaluate PHHs as a model for sex-specific in vitro research. In order to provide physiological culturing conditions, the added sex hormones were applied in concentrations that correspond to human serum levels. Compared to hormone concentrations that are used in common practice, ours were relatively low. This makes the analytics of some hormones using our applied LC-MS system challenging. As we observed in our LC-MS analyses, > 95% of the initially applied hormones were presumably metabolized after 24 h of cell culture. Although we could still observe hormone effects on gene expression in our RT-qPCR results, the extents were rather low. Nonetheless, we would not advocate the use of supraphysiological hormone concentrations as this would also not provide an appropriate physiological setting. The utilization of flow systems or at least shorter time intervals between media changes could offer improvements. Admittedly, this would increase the already high expense in time and costs of primary cell culture. However, our analytical spectrum provides information only on transcript level but does not go beyond.

In the context of hepatic sexual dimorphism and future in vitro research our study still provides valuable and novel insights. Previous transcriptomics studies have identified an advantageous situation for females concerning the expression of hepatic lipid metabolism genes that supports the clinical observation of premenopausal females being protected from MASLD. Our results did not confirm such a distinct sex bias in the analyzed genes. Nonetheless, sex-specific differences were observed and thus confirmed. Although in the surprising case of *LDLR* the direction was opposite to the literature report. In addition, our study provides further evidence that the sex of cells matters in in vitro research and that also the sex-specific environment in form of sex hormones has to be accounted for. In the context of MASLD research, our model can contribute to unravel a small part of the complex pathophysiological interactions, but takes not the complexity of a whole organ let alone systemic influences into account. In order to acquire a better understanding of the sex- and sex hormone-dependent mechanisms during hepatic steatosis, further studies that utilize PHHs of different sex in an in vitro steatosis model will follow.

## Conclusions

We have shown that the mRNA expression levels of *LDLR* are higher in male than female PHHs and that 17β-estradiol treatment decreased *LDLR* expression in PHHs of male donors. Further hepatic lipid metabolism genes were influenced by sex hormone treatment in PHHs of only one sex (e.g. *PPARA*). Thus, the sex-specific origin of primary cells and the hormonal environment these are cultivated in, should be taken into account in future research, especially when investigating pathophysiologies that show a sexual dimorphism as in the case of MASLD.

### Electronic supplementary material

Below is the link to the electronic supplementary material.

Supplementary Material 1

## Data Availability

The datasets used and/or analysed during the current study are available from the corresponding author on reasonable request.

## Abbreviations

ABCA1: ATP-binding cassette, sub-family A, member 1
APOA5: Apolipoprotein A-V
APOL2: Apolipoprotein L2
BMI: Body mass index
CPT2: Carnitine palmitoyltransferase 2
CRLM: Colorectal liver metastasis
DMSO: Dimethyl sulfoxide
ESI: Electrospray ion source
FBS: Fetal bovine serum
FNH: Focal nodular hyperplasia
iCCA: Intrahepatic cholangiocellular carcinoma
LC-MS: Liquid chromatography–mass spectrometry
LDLR: Low density lipoprotein receptor
LIPC: Hepatic lipase
MASLD: Metabolic dysfunction-associated steatotic liver disease
NAFLD: Non-alcoholic fatty liver disease
NET: Neuroendocrine tumor
PBS: Phosphate buffered saline
PHHs: Primary human hepatocytes
PLA1A: Phospholipase A1 member A
PPARA: Peroxisome proliferator-activated receptor alpha
UPLC: Ultra performance liquid chromatography
WME: William’s Medium E
