# Supplementary material for: Sex hormones differently regulate lipid metabolism genes in primary human hepatocytes
Source: BMC Endocr Disord. 2024 Aug 1;24:135. doi: 10.1186/s12902-024-01663-9 (PMC11292922; doi:10.1186/s12902-024-01663-9)
Supplement: Supplementary file 1 — Supplementary Material 1 [file 12902_2024_1663_MOESM1_ESM.pdf]

Additional file 1

Sex hormones differently regulate lipid metabolism genes in primary human hepatocytes

Lena Seidemann[1,2], Clara Paula Lippold[1,2], Carolin Marie Rohm[1,2], Julian Connor Eckel[1,2], Gerda Schicht[1,2], Madlen Matz-Soja[3], Thomas Berg[3], Daniel Seehofer[1], Georg Damm[1,2]*

[1]Department of Hepatobiliary Surgery and Visceral Transplantation, Clinic for Visceral, Transplant, Thoracic and Vascular Surgery, Leipzig University Medical Center, 04103 Leipzig, Germany

[2]Saxonian Incubator for Clinical Translation (SIKT), Leipzig University, 04103 Leipzig, Germany

[3]Division of Hepatology, Department of Medicine II, Leipzig University Medical Center, 04103 Leipzig, Germany

*Correspondence: georg.damm@medizin.uni-leipzig.de

Table S1 Donor data for PHH suspension cultures

Donor	Sex	Age	BMI [kg/m^2]	Steatosis[1]	Diagnosis
FD8	Female	44	22	None	Hemangioma
FD9	Female	46	27	None	Hemangioma
FD10	Female	48	22	None	PLD
MD8	Male	76	24	5%	iCCA
MD9	Male	59	26	None	Echinococcosis
MD10	Male	63	32	8%	HCC

[1]as reported in the postoperative pathohistological examination
Abbreviations: BMI, body mass index; PLD, polycystic liver disease; iCCA, intrahepatic cholangiocellular carcinoma; HCC, hepatocellular carcinoma.

Table S2 Primer specifications

Gene	Assay ID[1] or primer sequence[2] (fwd / rev)
ABCA1	SBH0311739
CPT2	SBH0341291
LDLR	SBH0324029
PLA1A	SBH0212386
PPARA	SBH1220322
CYP3A5	SBH0140291
APOA5	tgggactacttcagccagac / cctcgctcccactcagag
APOL2	aggaccaagtgagcagagag / tgaccatgtgacttgcaagc
LIPC	gcaactctctcgaagccatg / tgctcccgggtaaaggtatg
UGT2B15	tcagtgtggacatcaggacc / tcgatccaggggcttcattg
SULT1A1	ctggagaagttcatggtcgg / gaggtagagaacagggtggg
RPL13A	cctggaggagaagaggaaagaga / ttgaggacctctgtgtatttgtcaa
EEF2	agaagctgtggggtgacag / gatcagctggcagaaggtg
RPS18	acatcgatgggcggcggaaa / ctcccgccctcttggtgaggt

[1]Primers purchased from Qiagen
[2]Primers purchased from Biomers

Table S3 UP-LC gradient program

Time [min]	Flow [ml/min]	A [%]	B [%]	Curve
0	0.3	98	2	0.6
1	0.3	80	20	0.6
5	0.3	60	40	0.6
7	0.3	30	70	0.6
9	0.3	20	80	0.6
10	0.3	98	2	0.6

A: water with 0.05 mM formic acid and 0.05 mM NH_4Cl and B: pure acetonitrile

Table S4 Spectrometry parameters (Xevo XS QTof)

Index	ESI+	ESI-
Desolvation Gas Flow (L/h)	800	800
Desolvation Temperature (°C)	550	550
Source Temperature (°C)	120	120
Capillary Voltage (kV)	3	2
Collision Energy (eV)	10	6
Mass accuracy (kDa)	+/- 0.001	

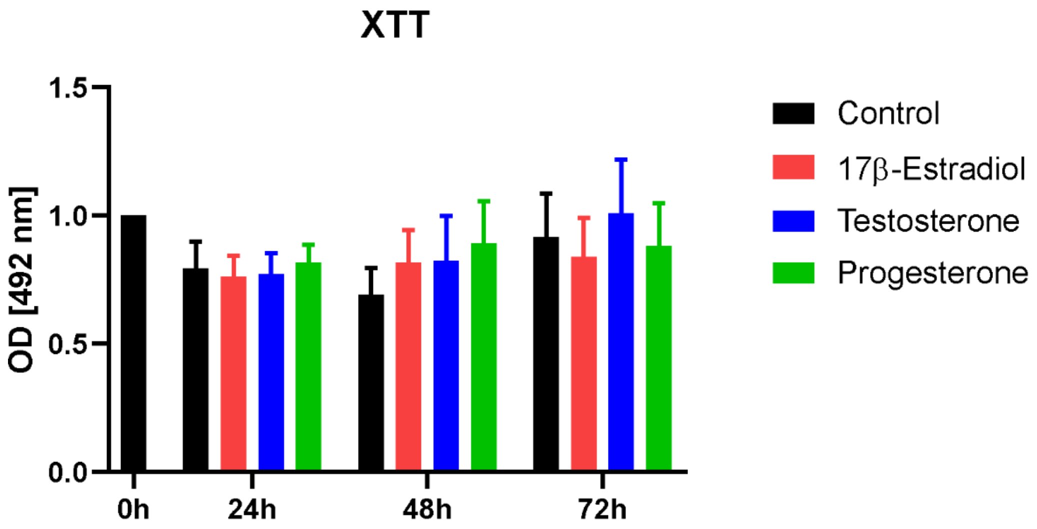

Fig. S1 Cell activity during cell culture with and without sex hormones was determined by colorimetric measurement of the conversion of XTT (2,3-bis-(2-methoxy-4-nitro-5-sulfophenyl)-2H-tetrazolium-5-carboxanilid). Data are normalized to the baseline value (0h) and are displayed as means + SEM; n = 7.

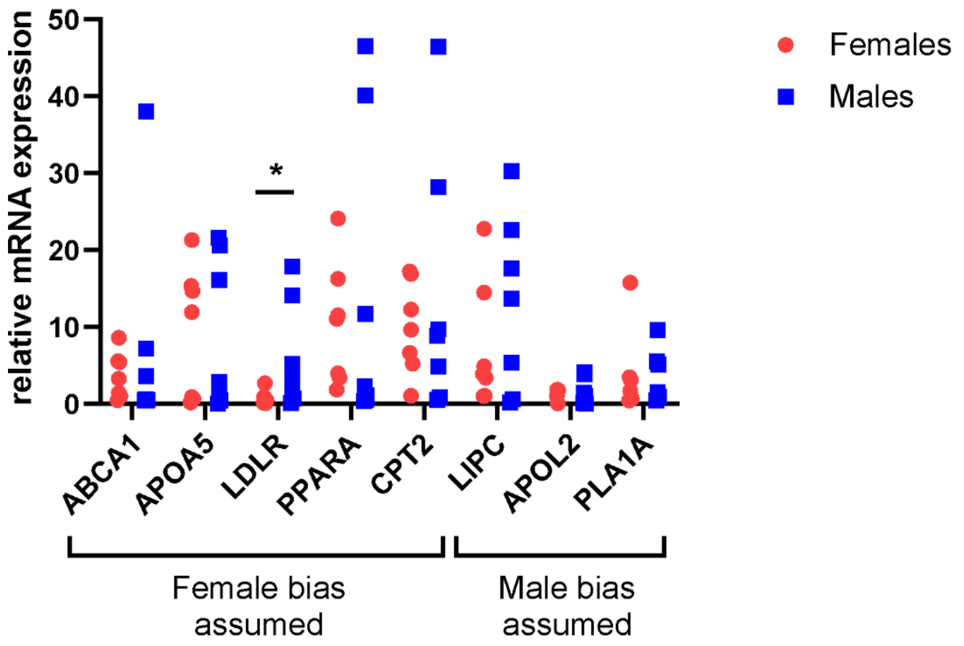

Fig. S2 Sex-specific mRNA expression levels of PHHs immediately post-isolation displayed as individual fold change values from female (dots) and male (boxes) donors. n = 7 per sex. *ABCA1*, ATP-binding cassette, sub-family A, member 1; *APOA5*, apolipoprotein A-V; *LDLR*, low density lipoprotein receptor; *PPARA*, peroxisome proliferator-activated receptor alpha; *CPT2*, carnitine palmitoyltransferase 2; *LIPC*, hepatic lipase; *APOL2*, apolipoprotein L2; *PLA1A*, phospholipase A1 member A.

Table S5 Sex-specific mRNA expression levels of PHHs immediately post-isolation

	**Relative mRNA expression in female PHHs**	**Relative mRNA expression in male PHHs**	**p Value**
ABCA1	2.42 (0.54)	1.76 (0.89)	0.691
APOA5	3.23 (1.03)	1.99 (1.32)	0.707
LDLR	0.36 (0.64)	2.45 (0.9)	**0.039**
PPARA	7.37 (0.47)	3.65 (1.03)	0.425
CPT2	7.39 (0.49)	5.75 (0.85)	0.741
LIPC	4.14 (0.6)	4.9 (1.05)	0.857
APOL2	0.5 (0.88)	0.67 (0.89)	0.805
PLA1A	3.42 (0.92)	4.26 (1.01)	0.834

Relative mRNA expression values are displayed as geometric means of individual fold change values (SEM).

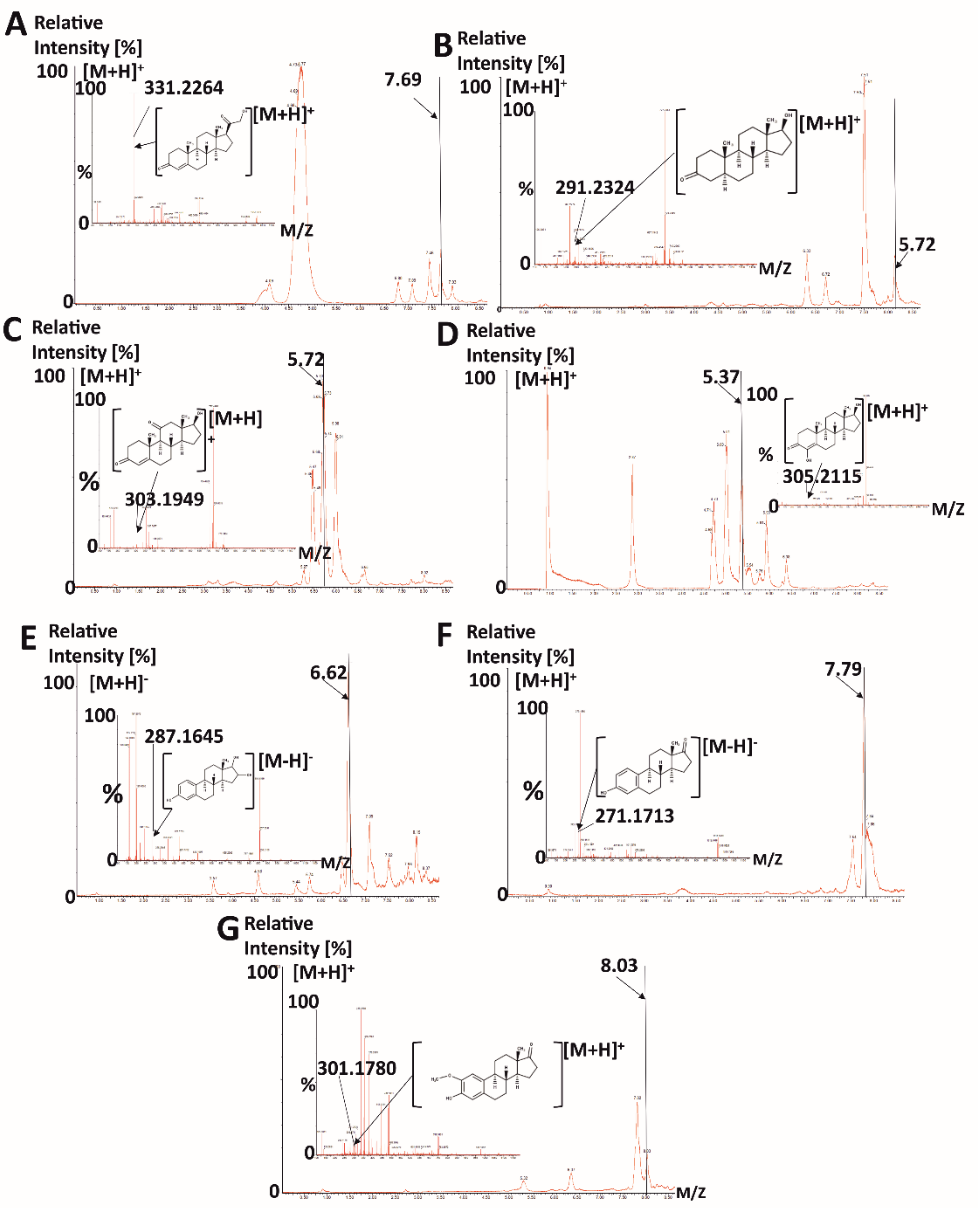

Fig. S3 Chromatograms and mass spectra of sex hormone metabolites. Primary human hepatocytes (PHHs) from female and male donors were cultured with 50 µM 17β-estradiol, testosterone or progesterone for 5 h. Cell culture media were collected and analyzed by LC-MS after cleavage of phase II metabolites. The following sex hormone metabolites were identified: **A** estrone, **B** 2-methoxyestrone, **C** 2-hydroxyestradiol, **D** hydroxytestosterone, **E** 11-ketotestosterone, **F** dihydrotestosterone, **G** hydroxyprogesterone.

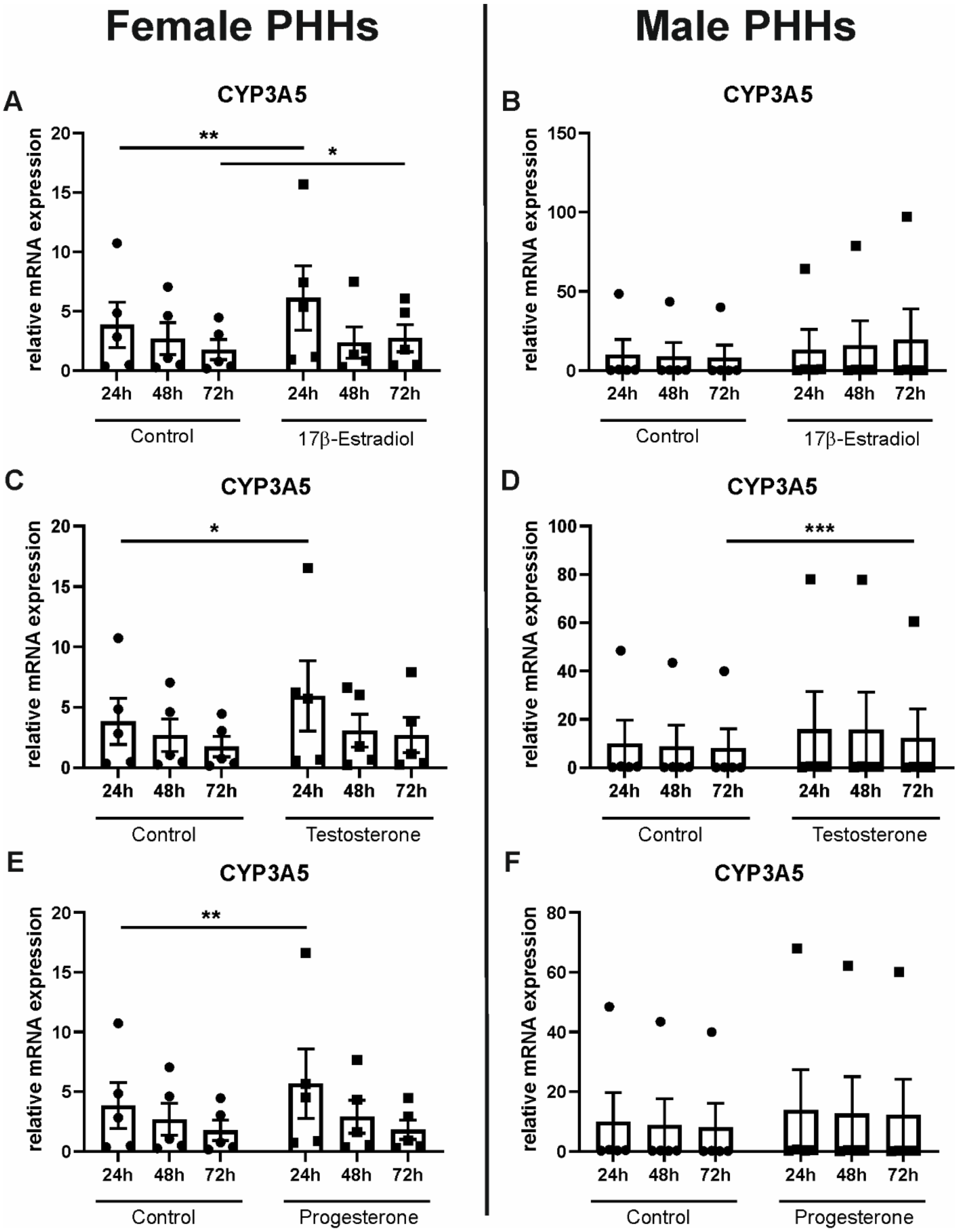

Fig. S4 Influence of 17β-estradiol, testosterone and progesterone on *CYP3A5* mRNA expression levels of primary human hepatocytes (PHHs) of different sex. PHHs were isolated from liver tissues of female (**A, C, E**) and male (**B, D, F**) donors, cultured with PHH starving medium supplemented with 10 nM 17β-estradiol (**A, B**), 40 nM testosterone (**C, D**) or 70 nM progesterone (**E, F**) for up to 72 h and mRNA expression levels of *CYP3A5* (Cytochrome P450 3A5) were analyzed by RT-qPCR. *RPL13A* (ribosomal protein L13a), *EEF2* (eukaryotic elongation factor 2) and *RPS18* (ribosomal protein S18) served as reference genes. Individual fold change values are displayed as dots and cubes, bar graphs display means ± SEM, n = 5 per sex, paired t test, $p < 0.05$ (*), $p < 0.0021$ (**), $p < 0.0002$ (***).

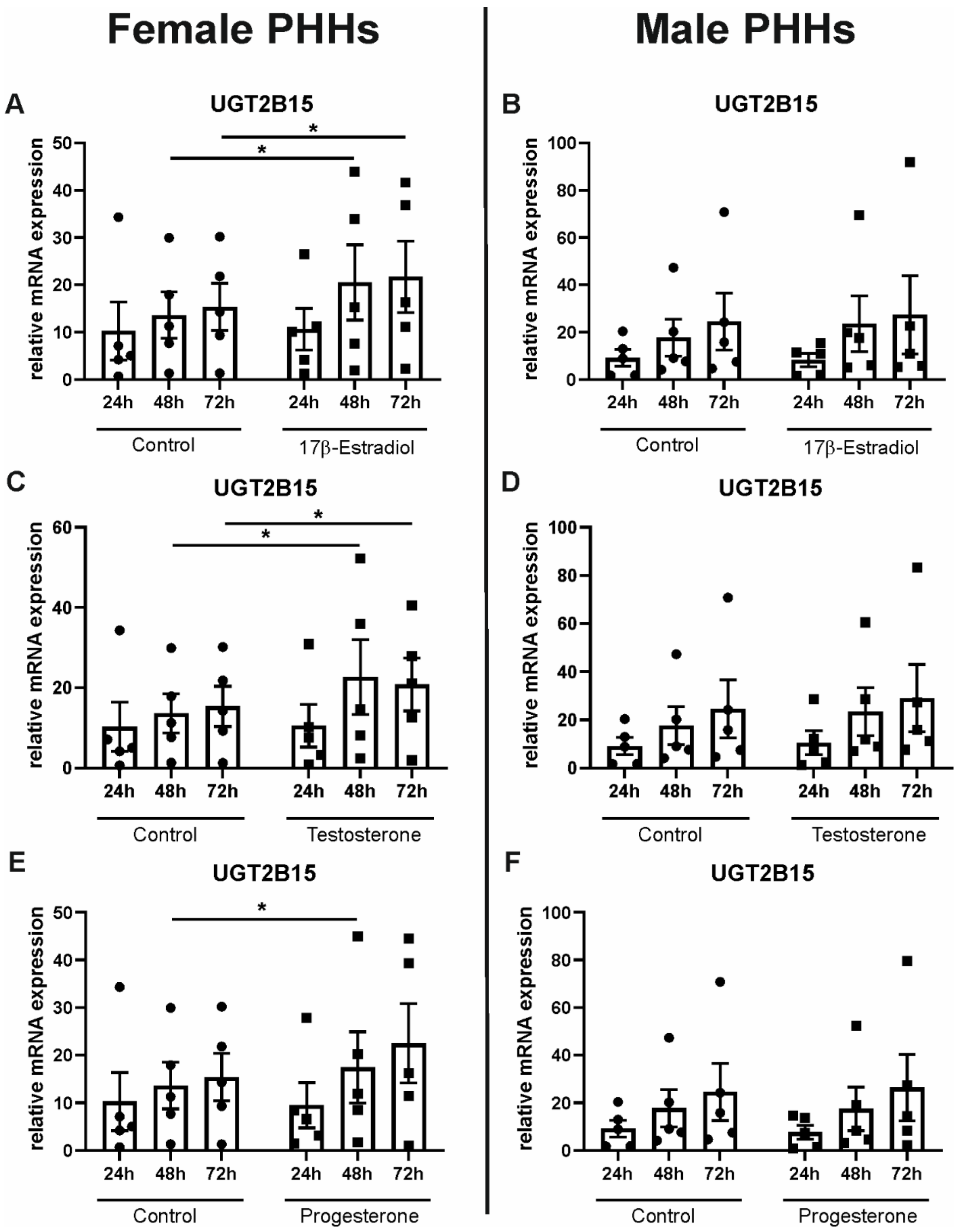

Fig. S5 Influence of 17β-estradiol, testosterone and progesterone on *UGT2B15* mRNA expression levels of primary human hepatocytes (PHHs) of different sex. PHHs were isolated from liver tissues of female (**A, C, E**) and male (**B, D, F**) donors, cultured with PHH starving medium supplemented with 10 nM 17β-estradiol (**A, B**), 40 nM testosterone (**C, D**) or 70 nM progesterone (**E, F**) for up to 72 h and mRNA expression levels of *UGT2B15* (UDP-glucuronosyltransferase 2B15) were analyzed by RT-qPCR. *RPL13A* (ribosomal protein L13a), *EEF2* (eukaryotic elongation factor 2) and *RPS18* (ribosomal protein S18) served as reference genes. Individual fold change values are displayed as dots and cubes, bar graphs display means ± SEM, n = 5 per sex, paired t test, $p < 0.05$ (*).

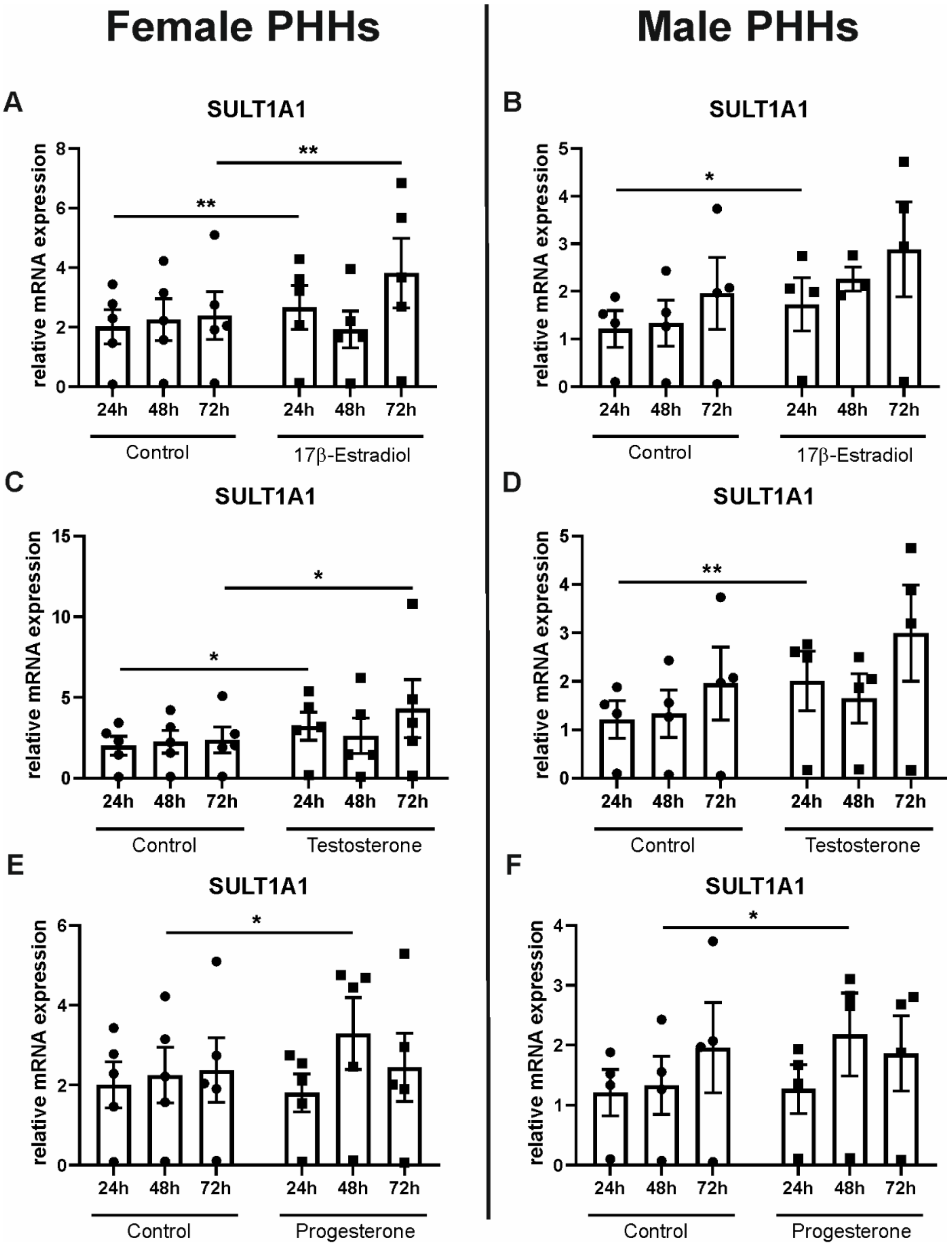

Fig. S6 Influence of 17β-estradiol, testosterone and progesterone on *SULT1A1* mRNA expression levels of primary human hepatocytes (PHHs) of different sex. PHHs were isolated from liver tissues of female (**A, C, E**) and male (**B, D, F**) donors, cultured with PHH starving medium supplemented with 10 nM 17β-estradiol (**A, B**), 40 nM testosterone (**C, D**) or 70 nM progesterone (**E, F**) for up to 72 h and mRNA expression levels of *SULT1A1* (sulfotransferase family 1A member 1) were analyzed by RT-qPCR. *RPL13A* (ribosomal protein L13a), *EEF2* (eukaryotic elongation factor 2) and *RPS18* (ribosomal protein S18) served as reference genes. Individual fold change values are displayed as dots and cubes, bar graphs display means ± SEM, n = 3-5 per sex, paired t test, $p < 0.05$ (*), $p < 0.0021$ (**).

Table S6 Effects of 17β-estradiol on cultured primary human hepatocytes (PHHs) of different sex

		Relative mRNA expression in female PHHs			Relative mRNA expression in male PHHs		
		Control	17β-estradiol	p value	Control	17β-estradiol	p value
ABCA1	24 h	0.7 (0.38)	0.57 (0.29)	ns	0.31 (1.37)	0.35 (1.8)	ns
	48 h	0.51 (0.26)	0.4 (0.19)	ns	0.34 (0.89)	0.13 (1.55)	ns
	72 h	0.5 (0.36)	0.42 (0.26)	ns	0.27 (0.72)	0.19 (1.07)	ns
APOA5	24 h	0.84 (0.89)	0.64 (0.59)	ns	0.68 (1.0)	0.71 (1.17)	ns
	48 h	0.46 (0.53)	0.2 (0.31)	ns	0.46 (0.33)	0.45 (0.91)	ns
	72 h	0.21 (0.23)	0.25 (0.2)	ns	0.21 (0.19)	0.26 (0.45)	ns
LDLR	24 h	0.43 (0.34)	0.3 (0.21)	ns	1.33 (2.39)	1.34 (2.08)	ns
	48 h	0.34 (0.2)	0.29 (0.32)	ns	1.36 (1.65)	1.12 (1.12)	ns
	72 h	0.37 (0.18)	0.27 (0.08)	ns	1.96 (1.8)	0.86 (1.03)	0.037
PPARA	24 h	3.53 (1.43)	6.08 (1.16)	ns	1.16 (3.92)	1.38 (6.08)	ns
	48 h	2.92 (1.38)	2.76 (0.97)	ns	1.69 (2.62)	1.5 (6.04)	ns
	72 h	2.36 (1.05)	3.23 (1.2)	0.011	1.47 (2.83)	1.64 (5.76)	ns
CPT2	24 h	2.52 (1.05)	2.29 (0.88)	ns	1.06 (1.71)	1.42 (1.98)	ns
	48 h	2.4 (1.0)	2.39 (0.76)	ns	1.75 (1.41)	0.98 (2.33)	ns
	72 h	2.26 (0.94)	2.12 (1.06)	ns	1.53 (1.52)	0.84 (2.27)	ns
LIPC	24 h	2.35 (1.78)	2.11 (1.33)	ns	1.44 (2.08)	1.23 (1.78)	ns
	48 h	3.28 (1.87)	3.02 (1.52)	ns	3.43 (3.49)	2.46 (3.49)	ns
	72 h	2.9 (1.37)	3.68 (1.48)	0.044	2.79 (3.38)	2.86 (3.17)	ns
APOL2	24 h	0.89 (0.64)	0.59 (0.38)	0.031	0.69 (0.92)	0.59 (0.82)	ns
	48 h	0.8 (0.5)	0.56 (0.29)	ns	1.0 (0.6)	0.55 (0.7)	ns
	72 h	0.82 (0.48)	0.71 (0.49)	ns	0.8 (0.6)	0.54 (0.74)	ns
PLA1A	24 h	0.9 (2.42)	0.74 (1.66)	ns	1.29 (0.88)	1.09 (0.52)	ns
	48 h	1.04 (4.68)	0.79 (4.49)	ns	1.37 (0.86)	1.2 (1.24)	ns
	72 h	1.52 (6.8)	1.26 (8.49)	ns	1.55 (1.18)	1.45 (2.17)	ns

Relative mRNA expression values are displayed as geometric means of individual fold change values (SEM).

Table S7 Effects of testosterone on cultured primary human hepatocytes (PHHs) of different sex

		**Relative mRNA expression in female PHHs**			**Relative mRNA expression in male PHHs**		
		Control	Testosterone	p value	Control	Testosterone	p value
ABCA1	24 h	0.7 (0.38)	0.59 (0.24)	ns	0.31 (1.37)	0.28 (1.57)	ns
	48 h	0.51 (0.26)	0.41 (0.17)	0.042	0.34 (0.89)	0.26 (1.11)	ns
	72 h	0.5 (0.36)	0.47 (0.29)	ns	0.27 (0.72)	0.27 (1.13)	ns
APOA5	24 h	0.84 (0.89)	0.52 (0.71)	ns	0.68 (1.0)	0.63 (1.29)	ns
	48 h	0.46 (0.53)	0.43 (0.61)	ns	0.46 (0.33)	0.71 (0.58)	ns
	72 h	0.21 (0.23)	0.25 (0.28)	ns	0.21 (0.19)	0.37 0.35)	0.031
LDLR	24 h	0.43 (0.34)	0.31 (0.19)	ns	1.33 (2.39)	1.06 (1.9)	ns
	48 h	0.34 (0.2)	0.28 (0.26)	ns	1.36 (1.65)	1.21 (1.47)	ns
	72 h	0.37 (0.18)	0.35 (0.39)	ns	1.96 (1.8)	1.48 (0.9)	ns
PPARA	24 h	3.53 (1.43)	3.9 (1.0)	ns	1.16 (3.92)	1.4 (5.76)	ns
	48 h	2.92 (1.38)	2.64 (1.34)	ns	1.69 (2.62)	3.08 (6.66)	ns
	72 h	2.36 (1.05)	3.54 (1.61)	ns	1.47 (2.83)	1.83 (5.34)	ns
CPT2	24 h	2.52 (1.05)	1.02 (0.55)	0.006	1.06 (1.71)	0.41 (0.75)	0.002
	48 h	2.4 (1.0)	0.86 (0.38)	0.0002	1.75 (1.41)	0.96 (1.14)	0.029
	72 h	2.26 (0.94)	1.12 (0.53)	0.011	1.53 (1.52)	0.57 (0.83)	0.002
LIPC	24 h	2.35 (1.78)	2.3 (1.4)	ns	1.44 (2.08)	1.1 (1.87)	ns
	48 h	3.28 (1.87)	3.54 (2.45)	ns	3.43 (3.49)	3.66 (3.48)	ns
	72 h	2.9 (1.37)	3.28 (1.89)	ns	2.79 (3.38)	3.37 (3.2)	ns
APOL2	24 h	0.89 (0.64)	0.64 (0.43)	ns	0.69 (0.92)	0.6 (0.94)	ns
	48 h	0.8 (0.5)	0.64 (0.4)	ns	1.0 (0.6)	0.7 (0.83)	ns
	72 h	0.82 (0.48)	0.77 (0.49)	ns	0.8 (0.6)	0.79 (0.63)	ns
PLA1A	24 h	0.9 (2.42)	0.88 (2.26)	ns	1.29 (0.88)	1.07 (0.79)	ns
	48 h	1.04 (4.68)	1.06 (6.72)	ns	1.37 (0.86)	2.09 (2.33)	ns
	72 h	1.52 (6.8)	1.13 (5.73)	ns	1.55 (1.18)	1.83 (1.48)	ns

Relative mRNA expression values are displayed as geometric means of individual fold change values (SEM).

Table S8 Effects of progesterone on cultured primary human hepatocytes (PHHs) of different sex

		**Relative mRNA expression in female PHHs**			**Relative mRNA expression in male PHHs**		
		Control	Progesterone	p value	Control	Progesterone	p value
ABCA1	24 h	0.7 (0.38)	0.6 (0.26)	ns	0.31 (1.37)	0.27 (1.84)	ns
	48 h	0.51 (0.26)	0.36 (0.19)	0.033	0.34 (0.89)	0.19 (0.89)	ns
	72 h	0.5 (0.36)	0.42 (0.21)	ns	0.27 (0.72)	0.18 (0.91)	ns
APOA5	24 h	0.84 (0.89)	0.34 (0.39)	0.031	0.68 (1.0)	0.5 (1.04)	ns
	48 h	0.46 (0.53)	0.4 (0.38)	ns	0.46 (0.33)	0.34 (0.51)	ns
	72 h	0.21 (0.23)	0.16 (0.22)	ns	0.21 (0.19)	0.26 (0.28)	ns
LDLR	24 h	0.43 (0.34)	0.25 (0.16)	0.021	1.33 (2.39)	1.13 (1.74)	ns
	48 h	0.34 (0.2)	0.33 (0.19)	ns	1.36 (1.65)	0.84 (1.1)	0.005
	72 h	0.37 (0.18)	0.24 (0.1)	0.033	1.96 (1.8)	0.87 (0.86)	ns
PPARA	24 h	3.53 (1.43)	3.77 (0.98)	ns	1.16 (3.92)	1.41 (6.6)	ns
	48 h	2.92 (1.38)	3.2 (1.25)	ns	1.69 (2.62)	1.61 (5.3)	ns
	72 h	2.36 (1.05)	3.28 (1.12)	0.047	1.47 (2.83)	1.64 (4.2)	ns
CPT2	24 h	2.52 (1.05)	1.02 (0.29)	0.003	1.06 (1.71)	0.47 (1.0)	0.0002
	48 h	2.4 (1.0)	0.87 (0.35)	<0.0001	1.75 (1.41)	0.61 (0.81)	0.0008
	72 h	2.26 (0.94)	0.76 (0.43)	0.0003	1.53 (1.52)	0.43 (0.81)	0.019
LIPC	24 h	2.35 (1.78)	1.89 (1.67)	0.021	1.44 (2.08)	1.0 (1.76)	0.035
	48 h	3.28 (1.87)	3.08 (1.87)	ns	3.43 (3.49)	2.61 (2.28)	ns
	72 h	2.9 (1.37)	2.9 (1.43)	ns	2.79 (3.38)	2.93 (3.22)	ns
APOL2	24 h	0.89 (0.64)	0.52 (0.32)	0.031	0.69 (0.92)	0.45 (0.9)	ns
	48 h	0.8 (0.5)	0.72 (0.4)	ns	1.0 (0.6)	0.64 (0.53)	0.009
	72 h	0.82 (0.48)	0.63 (0.42)	ns	0.8 (0.6)	0.62 (0.53)	ns
PLA1A	24 h	0.9 (2.42)	0.71 (1.81)	ns	1.29 (0.88)	0.95 (0.71)	ns
	48 h	1.04 (4.68)	0.97 (5.55)	ns	1.37 (0.86)	1.45 (0.93)	ns
	72 h	1.52 (6.8)	1.2 (5.87)	ns	1.55 (1.18)	1.73 (2.01)	ns

Relative mRNA expression values are displayed as geometric means of individual fold change values (SEM).
